# Evaluating the efficacy of aerobic exercise as therapy for depression and anxiety in women with PCOS: a systematic review

**DOI:** 10.1136/bmjsem-2025-002709

**Published:** 2026-01-19

**Authors:** Lorna Evelyn Mansell, Caitlin Fox-Harding, Robert U Newton, Pedro Lopez da Cruz, Sara Bayes, Favil Singh

**Affiliations:** 1 School of Medical and Health Science, Edith Cowan University, Joondalup, Western Australia, Australia; 2 Edith Cowan University, Exercise Medicine Research Institute, Joondalup, Western Australia, Australia; 3 School of Biomedical and Sports Science, Edith Cowan University, Joondalup, Western Australia, Australia; 4 Grupo de Pesquisa Em Exercício Para Populações Clínicas (GPCLIN), Universidade de Caxias do Sul, Caxias do Sul, Brazil; 5 Programa de Pós-Graduação em Ciências da Saúde, Universidade de Caxias do Sul, Caxias do Sul, Brazil; 6 School of Nursing and Midwifery, Edith Cowan University, Joondalup. Western Australia, Australia, Joondalup, Western Australia, Australia; 7 School of Nursing, Midwifery and Paramedicine, Australian Catholic University, Victoria, Australia, Joondalup, Western Australia, Australia

**Keywords:** Exercise, Depression, Anxiety, Women, Sports & exercise medicine

## Abstract

**Objective:**

This systematic review aims to evaluate the effectiveness of exercise interventions in alleviating depression and anxiety symptoms in women with polycystic ovary syndrome (PCOS).

**Data sources:**

PubMed, CINAHL, Cochrane Library, SportDiscus, EMBASE and Web of Science databases were searched from their inception to July 2025.

**Eligibility criteria for selecting studies:**

Randomised controlled trials were eligible if they examined the effects of exercise lasting ≥4 weeks on validated measures of depression and/or anxiety in women aged 18–45 with PCOS diagnosed using the Rotterdam criteria. Publications not written in English were excluded.

**Data extraction and synthesis:**

Two reviewers independently extracted data and assessed study quality using the Cochrane Risk of Bias tool. The review followed the Preferred Reporting Items for Systematic Reviews and Meta-Analyses and the Prisma in Exercise, Rehabilitation, Sport medicine and SporTs science guidelines. A narrative/qualitative synthesis was used to provide an overview of the current literature on the topic. Given the limited number of eligible trials, outcomes and measurement tools, a meta-analysis was not undertaken.

**Results:**

From 363 full-text records, three trials (n=221) met the inclusion criteria. Interventions lasted 12–16 weeks, and aerobic exercise (continuous, intermittent or high-intensity interval training) was prescribed at least three times per week. Across all studies, depression symptoms improved by 4.8%–32.4%, with one study indicating a minimal clinically important difference, while anxiety symptoms decreased by 3.6%–42.2%, measured using validated scales, including Hospital Anxiety and Depression Measurement Scale, Depression, Anxiety, and Stress Scale-21, Montgomery Åsberg Depression Rating Scale and Brief Scale for Anxiety.

**Conclusion:**

Findings should be interpreted cautiously due to limited report numbers, methodological concerns and heterogeneity in interventions and outcome measures. While this review aimed to assess all exercise modalities, only aerobic exercise interventions were identified. These interventions appear effective in reducing depression and anxiety symptoms in women with PCOS. Future research should include psychological outcomes and explore resistance or combined diet-exercise interventions.

**PROSPERO registration number:**

CRD42023408190.

**WHAT IS ALREADY KNOWN ON THIS TOPIC**

- Polycystic ovary syndrome (PCOS) is linked with higher rates of depression and anxiety.
- Exercise improves general mental health and quality of life in women with PCOS.

**WHAT THIS STUDY ADDS**

- This review focuses specifically on aerobic exercise effects on depression and anxiety from randomised trials.
- Despite a small number of studies, supervised aerobic exercises do have some positive effects in reducing depression and anxiety in this patient cohort.

**HOW THIS STUDY MIGHT AFFECT RESEARCH, PRACTICE OR POLICY**

- Findings support using aerobic exercise to reduce depressive and anxiety symptoms in PCOS and highlight the need for supervised and individually tailored interventions.

## Introduction

 Polycystic ovary syndrome (PCOS) is a prevalent endocrine disorder characterised by a complex interplay of physiological and psychological factors, leading to significant health implications for women. Despite its widespread occurrence, it is estimated that up to 70% of PCOS cases remain undiagnosed, particularly among women with higher body mass index (BMI).^1 2^ In Australia, approximately 1 in 10 women experiences PCOS during their reproductive years, often recognised as the leading cause of infertility in this demographic.^2 3^

The hallmark characteristics of PCOS include disruptions to the menstrual cycle, alterations in hair and skin patterns and the presence of polycystic ovary morphology, defined as multiple immature follicles on the ovaries, which indicates intricate hormonal imbalances.^1 4^ Beyond these physical manifestations, individuals with PCOS face heightened risks of developing chronic health issues such as cardiovascular disease, type II diabetes and metabolic syndrome.^2 5 6^ Crucially, the burden of PCOS extends beyond the somatic domain, as individuals are found to be 3–8 times more likely to deal with psychological symptoms such as depression and anxiety compared with their counterparts without the condition.^2 5 6^ The complexities of psychological symptoms in women with PCOS are influenced by a myriad of factors, including hormonal imbalances, body image concerns and the impact of chronic health issues. Addressing these psychological aspects is crucial for improving the overall quality of life (QOL) for individuals living with PCOS.^2 5^

Recognising the intricate relationship between physical and psychological symptoms of PCOS, comprehensive management strategies addressing both are imperative. While pharmacological interventions, lifestyle modifications (eg, improving dietary intake and increasing physical activity) and exercise medicine play pivotal roles in addressing the physical aspects of PCOS,^1 7^ the management of psychological symptoms remains a challenge. Traditional approaches, encompassing counselling and psychiatric interventions, have been employed to address the psychological ramifications of PCOS. However, the multifaceted nature of psychological challenges in PCOS necessitates a more integrated approach, encompassing both pharmacological and non-pharmacological interventions.^1 7 8^

International evidence-based guidelines for the management of PCOS now recommend lifestyle modification, including exercise, as first-line treatment across the lifespan.^1 7^ Exercise is well established as an adjunctive therapy for managing both physical and psychological aspects across a range of chronic conditions.^2 9 10^ For PCOS participants, exercise interventions have traditionally been implemented for physical benefits, including weight management and improvement in hormone levels; however, the potential impact on mental well-being remains an area warranting further exploration.^1 2 11^ Patten *et al*^12^ conducted a broad systematic review exploring how exercise interventions affect mental health and QOL in women with PCOS. Their review included a variety of study designs (randomised controlled trials (RCTs), single-arm and case–control), a mixed diagnostic criteria (Rotterdam and NIH), with depression and anxiety assessed as secondary outcomes. Building on this foundation, this review takes a more focused and methodological approach by including only RCTs that specifically assess depression and anxiety in women with PCOS diagnosed according to the Rotterdam criteria only. This allows for a more targeted understanding of how exercise may support mental health in this population. As a result, this review aims to synthesise the existing literature on the role of exercise in alleviating depression and anxiety associated with PCOS as the primary outcome.

## Method

All procedures undertaken in the review were reported in accordance with the Preferred Reporting Items for Systematic Reviews and Meta-Analyses (PRISMA) 2020 statement criteria,^13^ and reporting guideline,^14^ as well as the corresponding checklist.^15^ Checklist located in the electronic supplementary material 3. The PRISMA in Exercise, Rehabilitation, Sport medicine and SporTs science was also implemented.^16^ This review was prospectively registered with the International Prospective Register of Systematic Reviewers (PROSPERO identifier: CRD42023408190), with no protocol amendments.

### Eligibility criteria

This review included studies reporting on the impact of physical activity or exercise interventions on psychological symptom outcomes associated with PCOS. The inclusion criteria for this systematic review followed the Population, Intervention, Comparator, Outcomes, and Study design framework.^13^ Studies were included if (1) participants were diagnosed with PCOS using Rotterdam criteria^4^ and were within the reproductive years (aged 18–45 years); (2) any exercise and/or physical activity interventions lasting at least 4 weeks in duration; (3) investigating the effect of exercise and/or physical activity on depression, and/or anxiety; and (4) RCT with usual care control group of women diagnosed with PCOS. Publications not written in English were excluded. A 4-week minimum intervention duration was chosen to ensure sufficient exposure for psychological benefits. Tian *et al*^17^ found that exercise, regardless of type, was effective in reducing depressive symptoms after at least 4 weeks. Similarly, Blumenthal *et al*^18^ showed symptom reductions plateauing after week 4, reinforcing this threshold. Building on Patten *et al*,^12^ this review applies a more targeted criterion for clinical relevance.

### Information sources

A comprehensive search was conducted from inception to May 2023 using the following electronic databases: PubMed (via NLM), CINAHL (via EBSCOhost), Web of Science Core Collection, SPORTDiscus (via EBSCOhost), Cochrane Library and EMBASE (via Ovid). Grey literature was not included in this review, in accordance with the prespecified protocol. An updated search conducted in July 2025 found no new relevant studies.

### Search strategy

**Figure 1:**
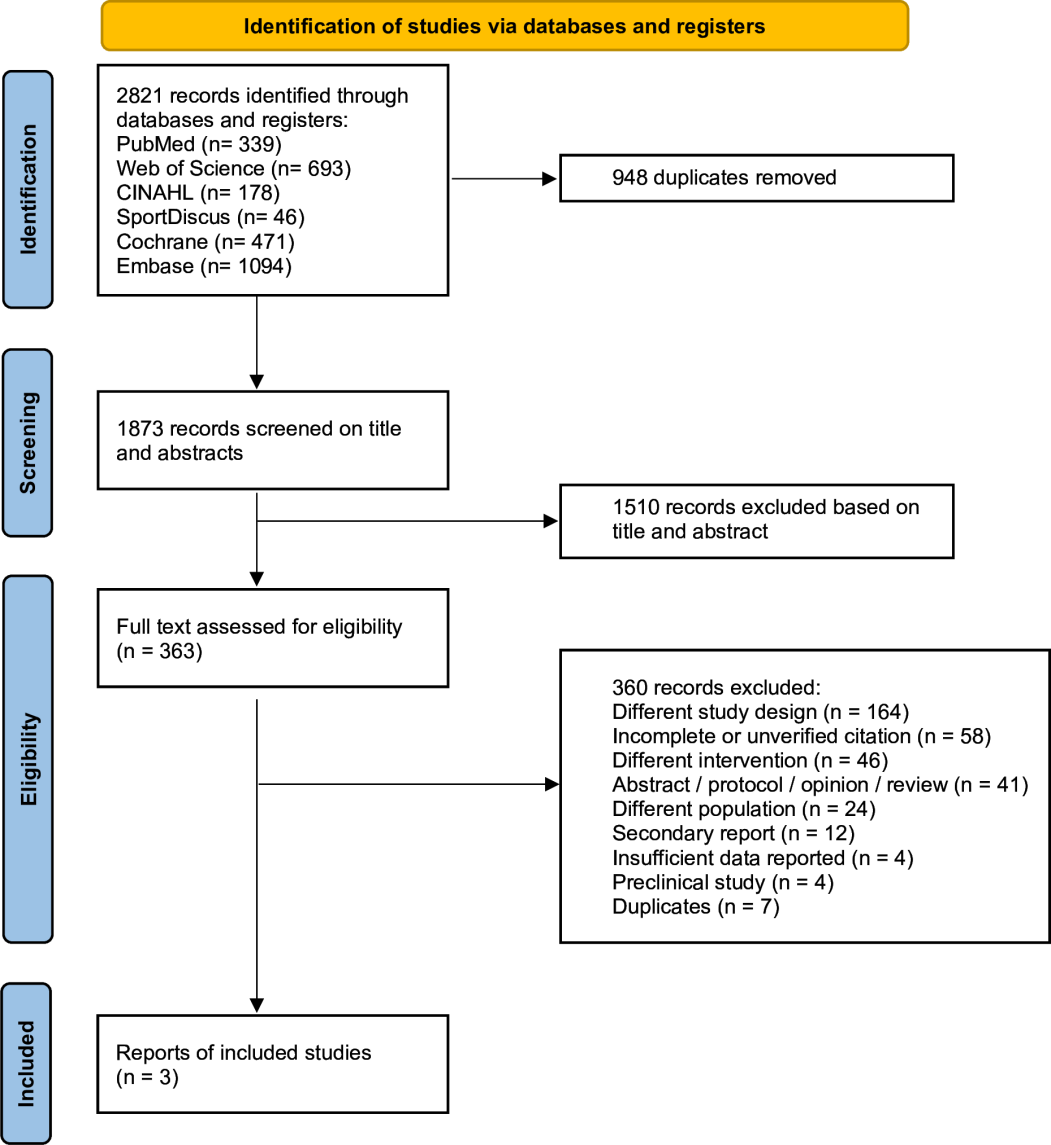
Flowchart of the study selection process. ‘Different’ indicates studies with unrelated designs, interventions or populations (eg, non-PCOS, non-exercise or non-psychological outcomes). ‘Insufficient data reported’ refers to studies lacking extractable or validated depression or anxiety outcome data. PCOS, polycystic ovary syndrome.

The search terms included a combination of keywords relating to PCOS, exercise, physical activity and RCTs. The search strategy was undertaken using controlled vocabulary and free-text terms as presented in figure 1. Relevant prior systematic reviews, including Patten *et al*^12^ were examined; however, as these focused broadly on mental health and QOL rather than depression and anxiety specifically, backward and forward citation tracking of included studies was performed to identify further eligible records.

### Selection process

Titles and abstracts were independently evaluated by two reviewers (LEM and FS) to screen records. Full‐text reports that met the criteria were retrieved and read independently by all reviewers and assessed for inclusion. Any disagreements were resolved through consensus, with a third reviewer (PLdC). A manual search of references in selected articles was performed to identify additional studies for inclusion. No automated tools were used throughout this process, and the authors of primary studies were not contacted to clarify eligibility.

### Data collection process

Two reviewers (LEM and PLdC) independently extracted data from the published texts and tables of the reports using the standardised form. The data extraction sheet is publicly available at OSF: https://osf.io/3ctdf/ and online supplementary tables 1 and 2. Discrepancies were resolved through discussion or adjudicated by a third reviewer (FS).

### Data items

The following data were extracted: (authors, year), sample characteristics (age, waist-to-hip ratio, BMI), PCOS characteristics, intervention design, control group type, exercise prescription (modality, frequency, intensity, duration), adherence, psychological and clinical outcomes, and statistical analysis methods. Additional data regarding power/sample size calculations and control group descriptions were also captured. Study-level details are summarised in table 1.

**Table 1:** Overview of characteristics of randomised controlled trials in women with PCOS

Study	Participants characteristics	Experimental design	Data analysis	PCOS characteristics	Outcomes
Kogure *et al*^21^	126 women from Brazil Age: 28.8±0.2 years BMI: 28.7±5.2 kg.m⁻² WHR: 0.8±0.0	Continuous aerobic (n=28) Intermittent aerobic (n=29) Control (n=30)* Supervised	Linear mixed-effects regression (between groups) Spearman correlation†	FAI: 9.5±3.2 Testosterone: 103.5±31.9 SHGB: 50.9±13.7	Anxiety Depression (HADS) Body image‡ Sexual function‡
Santos *et al*^23^	23 women from Brazil Age: 26.3±6.1 years BMI: 28.6±8.8 kg.m⁻² WHR: 0.8±0.1	HIIT training (n=12) Control (n=11)* Supervised	Shapiro-Wilk (normality) Independent t-test Fisher’s exact test (between groups) Cohen’s d (effect size)†	Absent menstrual cycle: 4.8±2.8	Anxiety Depression (DASS-21) Quality of life‡
Stener-Victorin *et al*^22^	72 women from Sweden Age: 29.9±4.4 years BMI: 28.1±7.4 kg.m⁻² WHR: 0.8±0.1	Aerobic training (n=29) Control (n=15)* Unsupervised	Kruskal-Wallis Mann-Whitney U (between groups) Wilcoxon signed-rank (within groups)†	Absent menstrual cycle: 4.0±1.1 Body hair: 3.4±2.0 Infertility: 4.0±1.7	Depression‡ (MADRS-S) Anxiety‡ (BSA-S) Quality of life PCOS symptoms

* Control groups varied across studies and included no intervention, brief advice or general physical activity information.

† Sample size calculation and/or normality testing were not reported in the original study.

‡ Primary outcomes. Data are expressed as mean±SD.

BMI, body mass index; BSA-S, Brief Scale for Anxiety; DASS-21, Depression, Anxiety, and Stress Scale; FAI, free androgen index; HADS, Hospital Anxiety and Depression Measurement Scale; MADRS-S, Montgomery Åsberg Depression Rating Scale; PCOS, polycystic ovary syndrome; SHBG, sex-hormone binding globulin; WHR, waist hip ratio.

### Study risk of bias assessment

Two reviewers (LEM and FS) assessed risk of bias using the Cochrane Risk of Bias tool, with each assessment performed at the outcome level for depression and anxiety symptoms (see online supplemental tables 1 and 2).^19 20^ Discrepancies were resolved through mutual agreement or with a third reviewer (PLdC). Due to the small number of included reports, formal assessment of publication bias through statistical tests was not performed. However, potential selective outcome reporting was considered by comparing reported outcomes with those prespecified in study protocols or trial registries when available.

### Effect measures

Depression and anxiety were the primary outcomes of this review. Mean and dispersion values were extracted from baseline and postassessment time points. If the reviewers of the identified reports did not include dispersion values of change, the SD of the change was calculated assuming a correlation of r=0.5 between the baseline and post-intervention assessment measures.^19 20^

### Synthesis methods

A narrative/qualitative synthesis was the preferred method to provide an overview of the current literature on the topic. Given the limited number of eligible trials, outcomes and measurement tools, a meta-analysis was not undertaken. Information extracted from studies included participant demographics, exercise/physical activity levels and exercise intervention information (frequency, intensity, time, type) as well as information on PCOS, depression and anxiety levels and body composition. When available, descriptive data as mean, median and dispersion values (eg, SD, 95% CIs) were reported.

## Results

### Study selection

The database searches identified 2821 records based on the search strategy. After removing 948 duplicates, 1873 studies were screened. Of these, a total of 1510 records were excluded based on titles and abstracts due to their irrelevance to the research question, resulting in 363 articles that were eligible for full-text screening. A total of 360 full-text reports were excluded (figure 1, online supplementary table 3). After a comprehensive assessment, three studies were included in this review.^21^,^23^

### Study characteristics

A total of 221 women with PCOS, with a median age of 28.7 years (IQR=25.1–31.5 years), were included in this review. The median BMI was 28.5 kg⁄m^2^ (IQR 24.3–32.7); with one study reporting an average hip-to-waist ratio of 0.82. Most participants were either overweight (n=177, 80.1%) or obese (n=44, 19.9%). The number of participants varied from 23 to 126 women across the included studies. Only two studies^21 23^ investigated supervised exercise interventions. Geographically, two studies^21 23^ were conducted in Brazil, while the third^22^ was conducted in Sweden. Santos *et al*^23^ reported that 40% of the participants were married and 66.7% had postsecondary education backgrounds (table 1).

### Clinical characteristics and assessments

The studies observed a range of PCOS-related characteristics. Kogure *et al*^21^ reported a mean free androgen index of 9.5, testosterone levels of 103.5 nmol/L and sex hormone binding globulin levels of 50.9 nmol/L. Santos *et al*^23^ found that 60% of participants experienced menstrual irregularities, with 50% reporting amenorrhoea and 10% reporting oligomenorrhoea. Stener-Victorin *et al*^22^ reported a mean score of 4.0±1.1 for menstrual problems on the PCOSQ scale, which ranges from 1 to 7, where higher scores indicate better QOL.^24^ Additionally, Stener-Victorin *et al*^22^ observed body hair scores of 3.4±2.0 and infertility scores of 4.0±1.7, further highlighting the impact of PCOS symptoms on daily functioning.

### Intervention characteristics

In the included studies, aerobic training was the sole intervention investigated (online supplemental table 2), with continuous, intermittent and high-intensity interval training (HIIT) being used. Specifically, Kogure *et al*^21^ examined the effects of both continuous and intermittent aerobic training, while Santos *et al*^23^ focused on a HIIT. Stener-Victorin *et al*^22^ explored self-selected aerobic training, where participants chose an exercise (eg, cycling, jogging, brisk walking) at an intensity described as ‘faster than normal walking’ for at least 30 min and at least 3 days a week.

Regarding the duration and frequency of the exercise sessions (online supplemental table 2), all studies had participants engaged in three sessions a week, with each session lasting 30–60 min. For the length of the exercise programme, Kogure *et al*^21^ and Stener-Victorin *et al*^22^ conducted their interventions for 16 weeks, while Santos *et al*^23^ had a 12-week intervention period.

The intensity of the training varied across the studies (online supplemental table 2). Kogure *et al*^21^ implemented a training programme with an intensity of 65%–80% of maximum heart rate (HRmax). Santos *et al*^23^ used a HIIT protocol with sessions at 95% of HRmax. Stener-Victorin *et al*^22^ required participants to maintain their heart rates greater than 120 bpm, approximately 70%–80% of HRmax. Adherence to the interventions, however, was not reported in any of the three studies.

### Risk of bias in studies

The quality assessment is detailed in online supplemental tables 1 and 2. Risk of bias was assessed at the outcome level for depression and anxiety. One study^21^ had a high risk of bias, primarily due to incomplete outcome data and high attrition rates, with imbalanced dropouts that were not adequately addressed. Additionally, there were uncertainties in the randomisation process and deviations from the intended interventions, raising concerns about selection bias. Another study^22^ was judged to have some concerns, primarily due to selection bias issues with the randomisation process. There were potential inadequacies in the sequence generation and allocation concealment. The third study^23^ had a low risk of bias across all categories.

### Outcomes

The included studies^21^,^23^ primarily measured depression and anxiety using various validated questionnaires, such as the Hospital Anxiety and Depression Scale (HADS), Depression, Anxiety and Stress Scale (DASS-21), Montgomery Åsberg Depression Rating Scale (MADRS-S) and Brief Scale for Anxiety (BSA-S).^25^,^28^ All studies measured BMI, with Kogure *et al*^21^ and Stener-Victorin *et al*^22^ randomising participants based on this variable. Only Kogure *et al*^21^ reported BMI outcomes and found no significant changes. No study directly reported formal diagnoses of depression/anxiety, diet, physical fitness, sleep or changes in medication use.

### Depression

Kogure *et al*^21^ utilised the HADS^25^ to assess symptoms of depression. A HADS-D score of 9 or more is considered high risk for depression. In the continuous aerobic training group, baseline HADS-D scores were 7.1 (SD=4.0), which significantly improved to 4.8 (SD=3.8) poststudy, representing a 32.4% improvement in depression. This 2.3-point reduction falls within the 1.2–3.3 minimal clinically important difference (MCID) range identified by Longo *et al*.^29^ Similarly, the intermittent aerobic training group showed a 28.6% improvement, with baseline scores decreasing from 5.6 (SD=3.6) to 4.0 (SD=3.2) poststudy. This 1.6-point reduction exceeds the 1.2 MCID threshold; however, it remains below the upper range of clinically significant change. Both exercise modalities led to clinically significant reductions (p=0.02) in depression scores following 16 weeks of intervention compared with control group.

Santos *et al*^23^ employed the DASS-21^26^ to evaluate the severity of depression. The DASS-21 consists of 21 questions, with seven questions specifically assessing depression. The scores are then categorised as: normal (0–9), mild (10–13), moderate (14–20), severe (21–27) and extremely severe (28+). HIIT baseline depression scores improved from 6.7 (SD=1.6) to 3.8 (SD=0.9), showing a 43.3% improvement postintervention. The intervention group showed a significant change (p=0.031) in comparison to the control group, which increased by 19.6%. A 30-day follow-up showed maintenance of depression scores of 3.8 (SD=0.9), while the control group increased depression by 24.6%.

Stener-Victorin *et al*^22^ used the MADRS-S^27^ to assess depression levels. The MADRS-S includes nine items rated on a 7-point Likert scale, where 0 indicates no symptoms and 6 indicates an extremely pathological condition, with ratings summed up to a maximum value of 54. A symptom burden of clinical relevance was defined as a total of more than 11. Over the 16 weeks of intervention, no significant changes were observed in the exercise group, showing a 4.8% change at postintervention. Similarly, there was 11.1% change in the control group (online supplemental table 2); however, there was no significant difference between the groups. A 32-week follow-up showed the exercise training group worsened in symptoms by 5.1%, from baseline, while the control group deteriorated by 7.2%, with no significant difference between groups.

### Anxiety

In the Kogure *et al*’s^21^ study, anxiety was assessed using the HADS questionnaire.^25^ A HADS-A score of eight or more is considered high risk for anxiety. In the continuous aerobic training group, baseline HADS-A scores were 8.9 (SD=3.8), improving to 6.7 (SD=3.6) poststudy, reflecting a 24.7% improvement. Similarly, in the intermittent aerobic training group, baseline values improved from 7.6 (SD=3.8) to 5.8 (SD=2.7) poststudy, reflecting a 23.7% improvement. Both groups significantly reduced anxiety symptoms (p=0.02) following 16 weeks of intervention compared with control group.

In the study by Santos *et al*,^23^ anxiety levels were measured using the DASS-21^26^ questionnaire. The scores are categorised as: normal (0–7), mild (8–9), moderate (10–14), severe (15–19) and extremely severe (20+). Reported baseline scores were 6.4 (SD=1.6), which improved to 3.7 (SD=0.7) following 12 weeks of HIIT, reflecting a 42.2% improvement in anxiety symptoms. The HIIT intervention resulted in a significant improvement (p=0.025) over 12 weeks compared with control group. A 30-day follow-up reported maintenance of anxiety levels of 3.5 (SD=0.6), while the control group increased by 8%.

Stener-Victorin *et al*^22^ used the BSA-S,^28^ which includes nine items rated on a 7-point scale, with ratings summed to a maximum value of 54, with lower scores indicating less severe symptoms. In the exercise group, baseline BSA-S was 11.3 (SD=6.0), which then had a 3.6% change in anxiety symptoms at 16 weeks and to 12.5% at 32 weeks. There was no significant change in anxiety scores in comparison to the control group at 16 weeks; however, there was a significant difference at 32 weeks (p=0.027).

## Discussion

This systematic review examined the role of exercise in enhancing psychological symptoms, focusing on depression and anxiety in women with PCOS. Findings were derived from three studies^21^,^23^ and showed that aerobic exercise prescribed at least three times per week, irrespective of whether it was continuous, intermittent or HIIT, reduced depression and anxiety levels in women diagnosed with PCOS. Intervention durations ranged from 12 to 16 weeks, with Kogure *et al*^21^ and Santos *et al*^23^ both incorporating supervised exercise 3x/week, which may have contributed to their outcomes, whereas the unsupervised, unstructured protocol used by Stener-Victorin *et al*^22^ yielded minimal effects.

From these findings, we suggest that aerobic exercise may alleviate a range of PCOS symptoms, including metabolic and hormonal issues, while also positively influencing psychological well-being.^11 30^ This review underscores the potential of aerobic exercise as a non-pharmacological intervention to improve psychological symptoms in women with PCOS. While broader reviews reported similar benefits,^9 10 12^ our findings suggest that intervention format (eg, supervision, intensity) may be critical, with only structured protocols demonstrating significant effects. Based on our findings, allied health providers may consider incorporating aerobic exercise into overall treatment plans to assist in addressing both physical and psychological aspects. It is important to address this issue, as visible symptoms of PCOS, such as changes in the menstrual cycle, hair and skin, and weight fluctuations, can negatively impact body image and potentially contribute to feelings of depression and anxiety.^21 31^ As evidenced by Kogure *et al*,^21^ exercise significantly improved body image, which may explain the significant reductions in depression and anxiety.^31^ Additionally, exercise not only enhances physical health but also boosts self-esteem and body satisfaction,^32^ helping to alleviate psychological symptoms. Therefore, exercise, tailored to the specific needs of women with PCOS, offers a holistic approach to managing the condition, promoting overall well-being by simultaneously improving body image and psychological health. This includes modifications in frequency, intensity and modality based on individual symptom severity, preferences and comorbidities, as recommended by the 2023 International PCOS guidelines.^1^

### Exercise benefits for depression and anxiety

Exercise interventions significantly improve depression scores across various populations,^9 10 33^ a benefit particularly relevant for women with PCOS. Physiologically, aerobic exercise enhances cardiovascular health, increases brain oxygenation and stimulates endorphin release, which can elevate mood.^34 35^ Activities such as running, cycling and swimming also build muscle endurance and flexibility, promoting overall physical well-being.^35^ The duration and intensity of aerobic sessions may be critical in optimising these benefits, with moderate-intensity exercise for at least 30 min on most days recommended for significant psychological improvements. Even modest changes may be clinically meaningful for women with PCOS.^2 11^

HIIT is another effective approach, as it alternates short bursts of intense activity with recovery periods, optimising both physiological and psychological benefits.^12 35^ Given the effectiveness in addressing both metabolic and mental health challenges,^23 35^ HIIT may be particularly beneficial for women with PCOS, improving overall health. This is largely attributed to its ability to stimulate key neuroendocrine responses associated with mood regulation and stress resilience. The higher intensity tends to produce greater endocrine responses, including endorphins, brain-derived neurotrophic factor, norepinephrine, dopamine, testosterone and growth hormone, all of which have a mechanistic influence to improve depression and anxiety.^34^,^36^ Despite not measuring endocrine response specifically in their study, Santos *et al*^23^ demonstrated similar psychological improvements using HIIT exercise protocol.

Beyond depression, exercise has shown promise in reducing anxiety symptoms.^9^ A report by Lara *et al*,^37^ 21% of participants with PCOS felt less anxious following 16 weeks of resistance training. Although Gordon *et al*^38^ examined healthy, young adults, their findings showed 20% reduction in anxiety symptoms after 8 weeks of resistance training, highlighting its broader psychological benefits. This supports the potential of resistance training to alleviate anxiety in women with PCOS, who are already at a higher risk of developing anxiety-related symptoms. Similarly, Rao *et al*,^39^ who reported a 41.8% reduction in anxiety symptoms following an 8-week aerobic exercise intervention in adults diagnosed with depression, further reinforces the mental health benefits of structured exercise.

Beyond its benefits, it is important to acknowledge that not everyone finds exercising a positive experience. Some experience anxiety about physical activity due to fears of injury, discomfort when exercising in front of others or physical limitations such as low physical fitness levels or medical concerns.^34^ Women with PCOS may not only share these concerns but also face additional fears, including doubts about whether exercise will meaningfully improve their symptoms, both metabolic and psychological.^1 7 34 40^

### Exercise benefits of supervised aerobic exercise

One potential factor to further ease women with PCOS who face exercise barriers is providing supervision assistance. Supervision during aerobic exercise interventions has shown to enhance physiological and psychological benefits, ensuring participants obtain maximal benefit from their efforts. This is particularly important for women with PCOS, who face unique challenges such as hormonal imbalances, increased fatigue and reduced exercise intolerance.^1 7^ They may benefit more from structured, supervised protocols like Santos *et al*^23^ (HIIT) compared with Stener-Victorin *et al*,^22^ whose self-selected, unsupervised aerobic exercise showed minimal improvements. From a physiological perspective, supervised exercise ensures that individuals receive immediate feedback to maintain correct form and technique, thereby maximising cardiovascular and muscular benefits while minimising the risk of injury.^41 42^ This is essential for achieving functional independence and long-term physical benefits, which are crucial factors for women with PCOS, who require long-term management of their condition.^41 43 44^ Proper guidance from trained professionals plays a vital role in this long-term management. It allows for tailored programmes that meet the individual needs in terms of intensity and duration, thus optimising improvements in their fitness levels and overall health, particularly in the context of managing PCOS symptoms.^43^ Furthermore, supervision and motivation encourage individuals to adhere to their exercise routines more consistently.^42 45^

This is particularly relevant in the context of managing psychological symptoms, where regular exercise is crucial for achieving therapeutic benefits.^33^ Beyond technical and motivational support, supervised exercise sessions can also create a supportive social environment, which has been shown to enhance psychological well-being. The social interaction and camaraderie that develop within supervised exercise groups can reduce feelings of isolation and provide emotional support, which are vital components in managing psychological symptoms and promote a sense of community and overall well-being.^42 45^ These themes are consistent with findings from qualitative research, which highlight the influence of psychological barriers, self-image and the importance of structured support in promoting exercise adherence among women with PCOS.^46^,^48^ Structured environments created by supervised sessions help individuals maintain a routine, which is particularly beneficial for those who may struggle with self-discipline or confidence in their abilities,^42 48^ thereby supporting sustained exercise and its psychological benefits in women with PCOS.

### Strengths and limitations

This review has notable limitations. Methodological concerns in the included studies—such as unclear randomisation, missing outcome data, small sample sizes and limited reporting of depression and anxiety as primary outcomes— constrain the strength of the evidence. The use of varied mental health questionnaires reduced comparability across studies, and heterogeneity in interventions, supervision levels and study designs prevented meta-analysis and precluded formal certainty assessment. While the inclusion criteria aimed to capture all relevant research, only three studies met the criteria, necessitating cautious interpretation of results.

Strengths of the review process included prospective registration, a comprehensive search strategy, independent duplicate screening and outcome-level risk of bias assessment. Limitations of the process include exclusion of grey literature and the inability to statistically synthesise findings due to study heterogeneity.

### Future directions

To address these gaps, future studies should prioritise examining both aerobic and resistance exercise interventions that address psychological symptoms in women with PCOS using RCTs. Resistance training, known for its benefits in muscle adaptation and hypertrophy,^2^ warrants further investigation for its potential psychological effects in women with PCOS. Standardised psychological assessments should be implemented to improve comparability, and supervised interventions may enhance adherence and consistency. Larger, high-quality RCTs are needed to strengthen the evidence base and facilitate meta-analysis to inform clinical recommendations.

## Conclusion

In conclusion, aerobic exercise may reduce depression and anxiety in women with PCOS, with supervised exercise interventions showing further benefits. Future research may consider prioritising psychological symptoms as primary outcomes and exploring the combined effects of exercise and dietary interventions, or resistance exercise alone. Additionally, addressing body image concerns is vital for enhancing overall well-being in this population. By focusing on these areas, allied health providers can develop more holistic treatment plans that address both physical and psychological aspects of PCOS, supporting improvements in depression and anxiety in women with PCOS.

## Supplementary material

10.1136/bmjsem-2025-002709 online supplemental file 1

10.1136/bmjsem-2025-002709 online supplemental file 2

10.1136/bmjsem-2025-002709 online supplemental file 3

10.1136/bmjsem-2025-002709 online supplemental material 1

## Data Availability

Data are available in a public, open access repository.
