# Supplementary material for: Evaluating the efficacy of aerobic exercise as therapy for depression and anxiety in women with PCOS: a systematic review
Source: BMJ Open Sport Exerc Med. 2026 Jan 19;12(1):e002709. doi: 10.1136/bmjsem-2025-002709 (PMC12820835; doi:10.1136/bmjsem-2025-002709)
Supplement: online supplemental file 1 [file bmjsem-12-1-s001.docx]

**Electronic Supplementary Material 1: tables and appendices.**

This file contains the core materials of the review, including the risk of bias assessments and comprehensive search strategies for all databases.

Table of Contents

[Table 1. 2](#_Toc213337702)

[Risk of bias table for anxiety. 2](#_Toc213337703)

[Table 2. 3](#_Toc213337704)

[Risk of bias table for depression. 3](#_Toc213337705)

[Appendix 1. 4](#_Toc213337706)

[Search Strategy for PubMed search. 4](#_Toc213337707)

[Appendix 2. 5](#_Toc213337708)

[Search Strategy for CINAHL, Cochrane Library, SportDiscus and Web of Science search. 5](#_Toc213337709)

[Appendix 3. 6](#_Toc213337710)

[Search strategy for EMBASE. 6](#_Toc213337711)

# Table 1.

## Risk of bias table for anxiety.

|  | Randomisation process | Deviations from the intended interventions | Missing outcome data | Measurement of the outcome | Selection of the reported result. | Overall |
| --- | --- | --- | --- | --- | --- | --- |
| Kogure et al. (2020) | 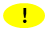 | 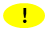 | 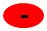 | 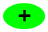 | 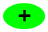 | 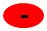 |
| Santos et al. (2022) | 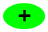 | 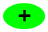 | 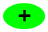 | 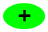 | 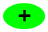 | 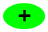 |
| Stener-Victorian et al. (2013) | 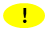 | 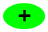 | 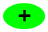 | 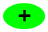 | 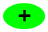 | 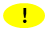 |

*Key:*
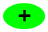
Low risk of bias,
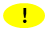
Unclear risk of bias,
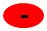
High risk of bias

# Table 2.

## Risk of bias table for depression.

|  | Randomisation process | Deviations from the intended interventions | Missing outcome data | Measurement of the outcome | Selection of the reported result. | Overall |
| --- | --- | --- | --- | --- | --- | --- |
| Kogure et al. (2020) | 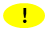 | 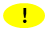 | 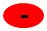 | 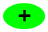 | 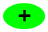 | 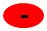 |
| Santos et al. (2022) | 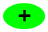 | 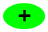 | 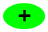 | 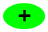 | 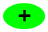 | 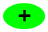 |
| Stener-Victorian et al. (2013) | 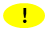 | 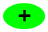 | 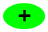 | 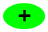 | 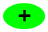 | 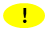 |

*Key:*
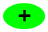
Low risk of bias,
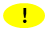
Unclear risk of bias,
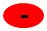
High risk of bias

# Appendix 1.

## Search Strategy for PubMed search.

(“Polycystic ovary syndrome” [title/abstract] OR “Polycystic ovarian syndrome” [title/abstract] OR PCOS [title/abstract] OR Ovary Syndrome, Polycystic [title/abstract] OR Syndrome, Polycystic Ovary [title/abstract] OR Stein-Leventhal Syndrome [title/abstract] OR Stein Leventhal Syndrome [title/abstract] OR Syndrome, Stein-Leventhal [title/abstract] OR Sclerocystic Ovarian Degeneration [title/abstract] OR Ovarian Degeneration, Sclerocystic [title/abstract] OR Sclerocystic Ovary Syndrome [title/abstract] OR Ovarian Syndrome, Polycystic [title/abstract] OR Polycystic Ovary Syndrome 1 [title/abstract] OR Sclerocystic Ovaries [title/abstract] OR Ovary, Sclerocystic [title/abstract] OR Sclerocystic Ovary) AND (exercise [title/abstract] OR Exercises [title/abstract] OR Physical Activity [title/abstract] OR Activities, Physical [title/abstract] OR Activity, Physical [title/abstract] OR Physical Activities [title/abstract] OR Exercise, Physical [title/abstract] OR Exercises, Physical [title/abstract] OR Physical Exercise [title/abstract] OR Physical Exercises [title/abstract] OR Exercise, Isometric [title/abstract] OR Exercises, Isometric [title/abstract] OR Isometric Exercises [title/abstract] OR Isometric Exercise [title/abstract] OR Exercise, Aerobic [title/abstract] OR Aerobic Exercise [title/abstract] OR Aerobic Exercises [title/abstract] OR Exercises, Aerobic [title/abstract] OR Exercise Training [title/abstract] OR Exercise Trainings [title/abstract] OR Training, Exercise [title/abstract] OR Trainings, Exercise [title/abstract] OR "resistance training" [title/abstract] OR "resistance exercise" [title/abstract] OR Training, Resistance [title/abstract] OR Strength Training [title/abstract] OR Training, Strength [title/abstract] OR Weight-Lifting [title/abstract] OR Strengthening Program [title/abstract] OR Strengthening Program, Weight-Lifting [title/abstract] OR Strengthening Programs, Weight-Lifting [title/abstract] OR Weight Lifting Strengthening Program [title/abstract] OR Weight-Lifting Strengthening Programs [title/abstract] OR Weight-Lifting Exercise Program [title/abstract] OR Exercise Program, Weight-Lifting [title/abstract] OR Exercise Programs, Weight-Lifting [title/abstract] OR Weight Lifting Exercise Program [title/abstract] OR Weight-Lifting Exercise Programs [title/abstract] OR Weight-Bearing Strengthening Program [title/abstract] OR Strengthening Program, Weight-Bearing [title/abstract] OR Strengthening Programs, Weight-Bearing [title/abstract] OR Weight Bearing Strengthening Program [title/abstract] OR Weight-Bearing Strengthening Programs [title/abstract] OR Weight-Bearing Exercise Program [title/abstract] OR Exercise Program, Weight-Bearing [title/abstract] OR Exercise Programs, Weight-Bearing [title/abstract] OR Weight Bearing Exercise Program [title/abstract] OR Weight-Bearing Exercise Programs [title/abstract] OR High Intensity Interval Training [title/abstract] OR High-Intensity Interval Trainings [title/abstract] OR Interval Training, High-Intensity [title/abstract] OR Interval Trainings, High-Intensity [title/abstract] OR Training, High-Intensity Interval [title/abstract] OR Trainings, High-Intensity Interval [title/abstract] OR High-Intensity Intermittent Exercise [title/abstract] OR Exercise, High-Intensity Intermittent [title/abstract] OR Exercises, High-Intensity Intermittent [title/abstract] OR High-Intensity Intermittent Exercises [title/abstract] OR Sprint Interval Training [title/abstract] OR Sprint Interval Trainings [title/abstract]) AND ("clinical trial" OR "randomized controlled trial" [title/abstract] OR "controlled clinical trial" [title/abstract] OR "randomized" [title/abstract] OR "randomly"[title/abstract] OR "groups" [title/abstract] OR "trial" [title/abstract])

# Appendix 2.

## Search Strategy for CINAHL, Cochrane Library, SportDiscus and Web of Science search.

(“Polycystic ovary syndrome” OR “Polycystic ovarian syndrome” OR PCOS OR Ovary Syndrome, Polycystic OR Syndrome, Polycystic Ovary OR Stein-Leventhal Syndrome OR Stein Leventhal Syndrome OR Syndrome, Stein-Leventhal OR Sclerocystic Ovarian Degeneration OR Ovarian Degeneration, Sclerocystic OR Sclerocystic Ovary Syndrome OR Ovarian Syndrome, Polycystic OR Polycystic Ovary Syndrome 1 OR Sclerocystic Ovaries OR Ovary, Sclerocystic OR Sclerocystic Ovary) AND (exercise OR Exercises OR Physical Activity OR Activities, Physical OR Activity, Physical OR Physical Activities OR Exercise, Physical OR Exercises, Physical OR Physical Exercise OR Physical Exercises OR Exercise, Isometric OR Exercises, Isometric OR Isometric Exercises OR Isometric Exercise OR Exercise, Aerobic OR Aerobic Exercise OR Aerobic Exercises OR Exercises, Aerobic OR Exercise Training OR Exercise Trainings OR Training, Exercise OR Trainings, Exercise OR "resistance training" OR "resistance exercise" OR Training, Resistance OR Strength Training OR Training, Strength OR Weight-Lifting OR Strengthening Program OR Strengthening Program, Weight-Lifting OR Strengthening Programs, Weight-Lifting OR Weight Lifting Strengthening Program OR Weight-Lifting Strengthening Programs OR Weight-Lifting Exercise Program OR Exercise Program, Weight-Lifting OR Exercise Programs, Weight-Lifting OR Weight Lifting Exercise Program OR Weight-Lifting Exercise Programs OR Weight-Bearing Strengthening Program OR Strengthening Program, Weight-Bearing OR Strengthening Programs, Weight-Bearing OR Weight Bearing Strengthening Program OR Weight-Bearing Strengthening Programs OR Weight-Bearing Exercise Program OR Exercise Program, Weight-Bearing OR Exercise Programs, Weight-Bearing OR Weight Bearing Exercise Program OR Weight-Bearing Exercise Programs OR High Intensity Interval Training OR High-Intensity Interval Trainings OR Interval Training, High-Intensity OR Interval Trainings, High-Intensity OR Training, High-Intensity Interval OR Trainings, High-Intensity Interval OR High-Intensity Intermittent Exercise OR Exercise, High-Intensity Intermittent OR Exercises, High-Intensity Intermittent OR High-Intensity Intermittent Exercises OR Sprint Interval Training OR Sprint Interval Trainings) AND ("clinical trial" OR "randomized controlled trial" OR "controlled clinical trial" OR randomized OR randomly OR groups OR trial)

# Appendix 3.

## Search strategy for EMBASE.

('ovary polycystic disease') AND (exercise OR 'physical activity' OR 'aerobic exercise' OR 'resistance training' OR 'weight machine' OR 'weight lifting' OR 'isometric exercise' OR 'weight bearing' OR 'high intensity interval training') AND ('clinical trial' OR 'randomized controlled trial' OR 'controlled study
