# Supplementary material for: Evaluating the efficacy of aerobic exercise as therapy for depression and anxiety in women with PCOS: a systematic review
Source: BMJ Open Sport Exerc Med. 2026 Jan 19;12(1):e002709. doi: 10.1136/bmjsem-2025-002709 (PMC12820835; doi:10.1136/bmjsem-2025-002709)
Supplement: online supplemental file 2 [file bmjsem-12-1-s002.docx]

**Electronic Supplementary Material 2: Data extraction and study exclusion.**

This file includes data extraction sheets for depression and anxiety, and a list of excluded studies at full-text screening.

Table of Contents

[Supplementary Table 1. 2](#_Toc213407584)

[Data extraction sheet for anxiety. 2](#_Toc213407585)

[Supplementary Table 2. 3](#_Toc213407586)

[Data extraction sheet for depression. 3](#_Toc213407587)

[Supplementary Table 3. 4](#_Toc213407588)

[Studies excluded at full-text screening with reasons (n=361) 4](#_Toc213407589)

# Supplementary Table 1.

## Data extraction sheet for anxiety.

# Supplementary Table 2.

## Data extraction sheet for depression.

# Supplementary Table 3.

## Studies excluded at full-text screening with reasons (n=361)

| **Year** | **Author** | **Title** | **Reason** | **More info** |
| --- | --- | --- | --- | --- |
| 2019 | Jiskoot, G.-//-Timman, R.-//-Beerthuizen, A.-//-De Loos, A. D.-//-Busschbach, J.-//-Laven, J. | The impact of a three-component lifestyle intervention on emotional well-being in women with PCOS | Abstract/Protocol/Opinion/Review | abstract only |
| 2007 | Ma, L. K.-//-Jin, L. N.-//-Yu, Q.-//-Xu, L. | [Effect of lifestyle adjustment, metformin and rosiglitazone in polycystic ovary syndrome] | Different study design | Abstract/Protocol/Opinion/Review |
| 2019 | Moran, L. J.-//-Stepto, N. K.-//-Brennan, L.-//-Garad, R.-//-Misso, M.-//-Norman, R.-//-Teede, H. | International evidence-based guideline for the assessment and management of polycystic ovary syndrome–Lifestyle management and models of care Guideline Development Group | Abstract/Protocol/Opinion/Review | duplicate |
| 2019 | Smith, C.-//-Bensoussan, A.-//-Arentz, S.-//-Abbott, J. | Herbal medicine plus lifestyle for overweight women with polycystic ovary syndrome: a randomised control trial | Duplicate | duplicate |
| 2017 | Arentz, S.-//-Smith, C. A.-//-Abbott, J.-//-Fahey, P.-//-Cheema, B. S.-//-Bensoussan, A. | Combined Lifestyle and Herbal Medicine in Overweight Women with Polycystic Ovary Syndrome (PCOS): A Randomized Controlled Trial | Duplicate | duplicate |
| 2019 | Arentz, S.-//-Smith, C.-//-Abbott, J.-//-Bensoussan, A. | Herbal medicine plus lifestyle for overweight women with polycystic ovary syndrome: A randomised control trial | Duplicate | duplicate |
| 2017 | Moran, L. J.-//-Brown, W. J.-//-McNaughton, S.-//-Joham, A. E.-//-Teede, H. J. | Weight management practices associated with polycystic ovary syndrome and their relationships with diet and physical activity | Duplicate | duplicate |
| 2020 | Oberg, E.-//-Lundell, C.-//-Blomberg, L.-//-Gidlof, S. B.-//-Egnell, P. T.-//-Hirschberg, A. L. | Psychological well-being and personality in relation to weight loss following behavioral modification intervention in obese women with polycystic ovary syndrome: a randomized controlled trial | Different intervention | interviews |
| 2022 | D'Souza, P.-//-Rodrigues, D. E.-//-Kaipangala, R. G.-//-Leena, K. C. | Effectiveness of Multimodular Interventions of Lifestyle Modification on Symptoms of Polycystic Ovarian Syndrome and Quality of Life among Women- A Quasi-experimental Study | Different study design | quality of life |
| 2011 | Ladson, G.-//-Dodson, W. C.-//-Sweet, S. D.-//-Archibong, A. E.-//-Kunselman, A. R.-//-Demers, L. M.-//-Williams, N. I.-//-Coney, P.-//-Legro, R. S. | The effects of metformin with lifestyle therapy in polycystic ovary syndrome: a randomized double-blind study | Different study design | quality of life |
| 2021 | Arentz, S.-//-Smith, C. A.-//-Abbott, J.-//-Bensoussan, A. | Perceptions and experiences of lifestyle interventions in women with polycystic ovary syndrome (PCOS), as a management strategy for symptoms of PCOS | Different intervention | survey |
| 2020 | Tay, C. T.-//-Moran, L. J.-//-Harrison, C. L.-//-Brown, W. J.-//-Joham, A. E. | Physical activity and sedentary behaviour in women with and without polycystic ovary syndrome: An Australian population-based cross-sectional study | Different intervention | survey |
| 2016 | Lim, A. J.-//-Huang, Z.-//-Chua, S. E.-//-Kramer, M. S.-//-Yong, E. L. | Sleep Duration, Exercise, Shift Work and Polycystic Ovarian Syndrome-Related Outcomes in a Healthy Population: A Cross-Sectional Study | Different intervention | survey |
| 2013 | Moran, L. J.-//-Ranasinha, S.-//-Zoungas, S.-//-McNaughton, S. A.-//-Brown, W. J.-//-Teede, H. J. | The contribution of diet, physical activity and sedentary behaviour to body mass index in women with and without polycystic ovary syndrome | Different intervention | survey |
| 2021 | Wright, P. J.-//-Corbett, C. L.-//-Pinto, B. M.-//-Dawson, R. M.-//-Wirth, M. D. | The impact of exercise perceptions and depressive symptoms on polycystic ovary syndrome-specific health-related quality of life | Different intervention | survey |
| 2022 | Sang, M.-//-Wu, Q.-//-Tao, Y.-//-Huang, F.-//-Lu, L.-//-Zhou, W.-//-Li, A.-//-Bai, S. | Usage of mobile health interventions among overweight/obese PCOS patients undergoing assisted reproductive technology treatment during the COVID-19 pandemic | Different intervention | survey |
| 2016 | Greenwood, E. A.-//-Noel, M. W.-//-Kao, C. N.-//-Shinkai, K.-//-Pasch, L. A.-//-Cedars, M. I.-//-Huddleston, H. G. | Vigorous exercise is associated with superior metabolic profiles in polycystic ovary syndrome independent of total exercise expenditure | Different intervention | survey |
| 2021 | Huang, D.-//-Jaswa, E.-//-Kao, C. N.-//-Quinn, M.-//-Cedars, M.-//-Huddleston, H. | Predictors of adequate physical activity within a multiethnic polycystic ovary syndrome patient population: a cross-sectional assessment | Different study design | survey |
| 2010 | Ctri | A clinical trial to evaluate the efficacy of yoga based lifestyle program on women with poly cycstic ovarian syndrome | Unverified citation | unlocated due to no author |
| 2020 | Ctri | A clinical trial to study the effect of exercise and metformin on mitochondrial health in patients with polycystic ovarian syndrome (PCOS) | Unverified citation | unlocated due to no author |
| 2016 | Nct | Adipose Tissue Function and Response to Exercise Training in Women With and Without Polycystic Ovary Syndrome | Unverified citation | unlocated due to no author |
| 2009 | Irct138902073819N | An evaluation of the efficacy of a "Home Base Exercise" program in clinical signs and hormonal and metabolic profile in patients with Poly Cystic Ovarian Syndrome (PCOS); a Randomized Clinical Trial | Unverified citation | unlocated due to no author |
| 2018 | Nct | An Integrated Self-Management Intervention for Adolescents With Polycystic Ovary Syndrome | Unverified citation | unlocated due to no author |
| 2017 | Actrn | An Intervention Program to Reduce the Risk Factors of Metabolic Syndrome in Malaysian Female University Staff with Polycystic Ovarian Syndrome | Unverified citation | unlocated due to no author |
| 2018 | Actrn | Do regular activity breaks from prolonged sitting improve the cardiometabolic profile of women with polycystic ovary syndrome? The PCOS BREAKS Study | Unverified citation | unlocated due to no author |
| 2022 | Nct | Effect of Aerobic Exercise on Polycystic Ovary Syndrome | Unverified citation | unlocated due to no author |
| 2020 | Irct20200722048167N | Effect of aerobic training and omega-3 exercise on oxidative and inflammatory elements in obese women | Unverified citation | unlocated due to no author |
| 2011 | Irct201104032892N | Effect of diet and exercise program to improve polycystic ovary syndrome (PCOS) | Unverified citation | unlocated due to no author |
| 2018 | Nct | Effect of Exercise on Cardiometabolic Profile in Women With Polycystic Ovary Syndrome | Unverified citation | unlocated due to no author |
| 2020 | db, R. B. R. | Effect of exercise training on clinical markers ,quality of life and mental health in women with polycystic ovary syndrome | Unverified citation | unlocated due to no author |
| 2022 | Nct | Effect of Periodized Resistance Training and High Intensity Training on BMI and QOL in PCOS | Unverified citation | unlocated due to no author |
| 2018 | Isrctn | Effect of physical exercise on treadmill in women with polycystic ovary syndrome | Unverified citation | unlocated due to no author |
| 2020 | Ctri | Effect of Pilates exercise on fat reduction and improving quality of life in women with Polycystic Ovarian Syndrome (PCOS) | Unverified citation | unlocated due to no author |
| 2014 | Actrn | Effect of progressive resistance training in women with polycystic ovary syndrome. A feasibility study | Unverified citation | unlocated due to no author |
| 2018 | jt, R. B. R. | Effect of the use of L-Arginine, Caffeine or Creatine supplements associated with physical activity in women with Polycystic Ovary Syndrome with Metabolic Syndrome | Unverified citation | unlocated due to no author |
| 2021 | Nct | Effect Of Treadmill Based Aerobic Exercise Intervention On Menstruation And Quality Of Life In Women With Polycystic Ovarian Syndrome | Unverified citation | unlocated due to no author |
| 2018 | Irct20180824040856N | Effect of trx training and omega-3 supplementation in controlling polycystic ovary syndrome | Unverified citation | unlocated due to no author |
| 2021 | Ctri | Effectiveness of intervention on physiological and biochemical parameters of Polycystic Ovarian syndrome | Unverified citation | unlocated due to no author |
| 2021 | Nct | Effects Of Combined Cryolipolysis And High Intensity Interval Training On Insulin Resistance And Body Composition In Polycystic Ovarian Patients | Unverified citation | unlocated due to no author |
| 2010 | Nct | Effects of Exercise for Overweight Women With Polycystic Ovary Syndrome | Unverified citation | unlocated due to no author |
| 2021 | Nct | Effects of High-Intensity Interval Training and Strength Training On Levels of Testosterone and Physical Activity Among Women With Polycystic Ovary Syndrome | Unverified citation | unlocated due to no author |
| 2018 | nhy6h, R. B. R. | Effects of physical exercise on cholesterol, glycemia and menstrual cycle of women with micropolicistos in the ovary | Unverified citation | unlocated due to no author |
| 2016 | qtwy, R. B. R. | Effects of treadmill physical exercise in women with polycystic ovaries | Unverified citation | unlocated due to no author |
| 2020 | Irct20200114046123N | Evaluation of the effects of adolescent health promotion training package on treatment of poly cystic ovary disease | Unverified citation | unlocated due to no author |
| 2021 | Ctri | Feasibility of diet and exercise as a treatment option for women with Polycystic ovarian syndrome who desire fertility: a preliminary study | Unverified citation | unlocated due to no author |
| 2015 | Nct | Graceful Lifestyle Changes Study for PCOS and Infertility | Unverified citation | unlocated due to no author |
| 2022 | Nct | High Intensity Resistance and Aerobic Training Among Women With PCOS | Unverified citation | unlocated due to no author |
| 2022 | Actrn | High-Intensity Functional Training for Polycystic Ovary Syndrome | Unverified citation | unlocated due to no author |
| 2018 | Nct | Homoeopathy and Yoga in the Treatment of Menstrual Disorders in Females With Polycystic Ovarian Syndrome | Unverified citation | unlocated due to no author |
| 2022 | Pactr | Impact of adding high probiotic food supplements to the diet and yoga on insulin resistance in polycystic ovarian syndrome | Unverified citation | unlocated due to no author |
| 2021 | Ctri | Impact of online lifestyle therapy on PCOS related symptoms in adolescent girls | Unverified citation | unlocated due to no author |
| 2015 | Nct | Improving Reproductive Function in Women With Polycystic Ovary Syndrome by High Intensity Interval Training | Unverified citation | unlocated due to no author |
| 2018 | Irct20161116030923N | Increasing physical activity among women with polycystic ovarian syndrome | Unverified citation | unlocated due to no author |
| 2022 | Nct | Lifestyle Change in Women With Polycystic Ovary Syndrome | Unverified citation | unlocated due to no author |
| 2011 | Nct | Lifestyle Intervention for Polycystic Ovary Syndrome: pulse-Based Diet and Exercise | Unverified citation | unlocated due to no author |
| 2023 | Ctri | ovary cyst and exercises | Unverified citation | unlocated due to no author |
| 2020 | Ctri | PHYTOESTROGEN RICH SUPPLEMENTATION ON MENSTRUAL IRREGULARITIES, PSYCHOLOGICAL STRESS AND PHYSICAL ACTIVITY LEVELS IN WOMEN WITH PCOS | Unverified citation | unlocated due to no author |
| 2017 | Nct | Polycystic Ovary Syndrome and Exercise | Unverified citation | unlocated due to no author |
| 2016 | qxgjh, R. B. R. | Polycystic Ovary Syndrome and Physical Exercise | Unverified citation | unlocated due to no author |
| 2009 | Nct | Short-term Structured Exercise Training Program Plus Diet Intervention in Patients With Polycystic Ovary Syndrome (PCOS) | Unverified citation | unlocated due to no author |
| 2007 | Nct | Structured Exercise Training Program Versus Hypocaloric Hyperproteic Diet in Obese Anovulatory Infertile Patients With PCOS | Unverified citation | unlocated due to no author |
| 2020 | Irct20130812014333N | Study of the effect of oats supplementation with exercise on anthropometric status, some metabolic and inflammatory profiles of women with polycystic ovary syndrome | Unverified citation | unlocated due to no author |
| 2015 | Actrn | The (i)mpact of (H)igh (I)ntensity intermittent (T)raining on health and mechanisms of insulin resistance in women with (P)oly(c)ystic (O)vary (S)yndrome: the iHIT-PCOS randomised control trial | Unverified citation | unlocated due to no author |
| 2012 | Actrn | The addition of naturopathic herbal medicine to a lifestyle intervention for women with polycystic ovary syndrome (PCOS), a randomised controlled trial | Unverified citation | unlocated due to no author |
| 2021 | Irct20211106052979N | The effect of aerobic training and DASH diet on insulin resistance and sex hormones in women with polycystic ovary syndrome | Unverified citation | unlocated due to no author |
| 2012 | Irct201212127255N | The effect of exercise in the treatment of polycystic ovary syndrome | Unverified citation | unlocated due to no author |
| 2018 | Zademodarres, S.-//-Motevasselian, M.-//-Bahramzade, S.-//-Amirnazari, B. | THE EFFECT OF HIGH INTENSITY INTERVAL TRAINING (HIT) ON INSULIN RESISTANCE AMONG WOMEN WITH POLYCYSTIC OVARIAN SYNDROME | Unverified citation | unlocated due to no author |
| 2022 | Irct20220404054405N | The effect of HIIT on endothelial function in PCOS | Unverified citation | unlocated due to no author |
| 2016 | Irct2016012326158N | The Effect OF Yoga on Quality Life, In Vitro Fertilisation Outcomes and Signs of Women With Poly Cystic Ovary Syndrom Undergoing Infertility Treatment | Unverified citation | unlocated due to no author |
| 2022 | Nct | The Effects of High-intensity and Moderate-intensity Exercise on Polycystic Ovary Syndrome | Unverified citation | unlocated due to no author |
| 2013 | Actrn | The health benefits of high intensity intermittent exercise in women with Polycystic Ovary Syndrome | Unverified citation | unlocated due to no author |
| 2020 | Nct | Yoga Therapy and Aerobic Exercise on Anti-Mullerian Hormone in Young Women With Polycystic Ovary Syndrome | Unverified citation | unlocated due to no author |
| 2005 | Euctr, D. K. | A randomised study of the effect of metformin and exercise in overweight women with polycystic ovary syndrome - Metformin-exercise project | Unverified citation | unlocated due to no author |
| 2017 | Bisgaard, H.-//-Dela, F. | [Physical exercise is a help for lean women with polycystic ovary syndrome] | Abstract/Protocol/Opinion/Review |  |
| 2014 | McBreairty, L.-//-Zello, G.-//-Rooke, J.-//-Serrao, S.-//-Pierson, R.-//-Chizen, D.-//-Chilibeck, P. | A pulse-based diet and exercise training in women with polycystic ovarian syndrome: effects on body composition, blood lipids and reproductive measures | Abstract/Protocol/Opinion/Review |  |
| 2017 | Jiskoot, G.-//-Benneheij, S. H.-//-Beerthuizen, A.-//-de Niet, J. E.-//-de Klerk, C.-//-Timman, R.-//-Busschbach, J. J.-//-Laven, J. S. | A three-component cognitive behavioural lifestyle program for preconceptional weight-loss in women with polycystic ovary syndrome (PCOS): a protocol for a randomized controlled trial | Abstract/Protocol/Opinion/Review |  |
| 2022 | Bazzi, A.-//-Schon, S. | Beyond diet and exercise: another option for patients with obesity and polycystic ovary syndrome? | Abstract/Protocol/Opinion/Review |  |
| 2023 | Kogure, G. S.-//-Lara, Lads-//-Ribeiro, V. B.-//-Lopes, I. P.-//-Mendes, M. C.-//-Kodato, S.-//-Ferriani, R. A.-//-Furtado, C. L. M.-//-Dos Reis, R. M. | Distinct Protocols of Physical Exercise May Improve Different Aspects of Well-being in Women With Polycystic Ovary Syndrome | Abstract/Protocol/Opinion/Review |  |
| 2020 | McBreairty, L. E.-//-Kazemi, M.-//-Chilibeck, P. D.-//-Gordon, J. J.-//-Chizen, D. R.-//-Zello, G. A. | Effect of a pulse-based diet and aerobic exercise on bone measures and body composition in women with polycystic ovary syndrome: A randomized controlled trial | Abstract/Protocol/Opinion/Review |  |
| 2006 | Moran, L. J.-//-Brinkworth, G.-//-Noakes, M.-//-Norman, R. J. | Effects of lifestyle modification in polycystic ovarian syndrome | Abstract/Protocol/Opinion/Review |  |
| 2013 | Orio, F.-//-Muscogiuri, G.-//-Ascione, A.-//-Marciano, F.-//-Volpe, A.-//-La Sala, G.-//-Savastano, S.-//-Colao, A.-//-Palomba, S. | Effects of physical exercise on the female reproductive system | Abstract/Protocol/Opinion/Review |  |
| 2022 | Sonntag, B. | Effects of training intensity on polycystic ovary syndrome markers: a randomized-controlled trial | Abstract/Protocol/Opinion/Review |  |
| 2018 | Jiskoot, G.-//-Benneheij, S.-//-Beerthuizen, A.-//-Timman, R.-//-Busschbach, J.-//-Laven, J. | Effects on body weight of a 1-year three-component lifestyle RCT in obese PCOS women | Abstract/Protocol/Opinion/Review |  |
| 2023 | Patten, R. K.-//-McIlvenna, L. C.-//-Moreno-Asso, A.-//-Hiam, D.-//-Stepto, N. K.-//-Rosenbaum, S.-//-Parker, A. G. | Efficacy of high-intensity interval training for improving mental health and health-related quality of life in women with polycystic ovary syndrome | Abstract/Protocol/Opinion/Review |  |
| 2020 | Moran, L. J.-//-Tassone, E. C.-//-Boyle, J.-//-Brennan, L.-//-Harrison, C. L.-//-Hirschberg, A. L.-//-Lim, S.-//-Marsh, K.-//-Misso, M. L.-//-Redman, L.-//-Thondan, M.-//-Wijeyaratne, C.-//-Garad, R.-//-Stepto, N. K.-//-Teede, H. J. | Evidence summaries and recommendations from the international evidence-based guideline for the assessment and management of polycystic ovary syndrome: Lifestyle management | Abstract/Protocol/Opinion/Review |  |
| 2012 | Costello, M. F.-//-Ledger, W. L. | Evidence-based lifestyle and pharmacological management of infertility in women with polycystic ovary syndrome | Abstract/Protocol/Opinion/Review |  |
| 2011 | Thomson, R. L.-//-Buckley, J. D.-//-Brinkworth, G. D. | Exercise for the treatment and management of overweight women with polycystic ovary syndrome: a review of the literature | Abstract/Protocol/Opinion/Review |  |
| 2019 | Stepto, N. K.-//-Patten, R. K.-//-Tassone, E. C.-//-Misso, M. L.-//-Brennan, L.-//-Boyle, J.-//-Boyle, R. A.-//-Harrison, C. L.-//-Hirschberg, A. L.-//-Marsh, K.-//-Moreno-Asso, A.-//-Redman, L.-//-Thondan, M.-//-Wijeyaratne, C.-//-Teede, H. J.-//-Moran, L. J. | Exercise Recommendations for Women with Polycystic Ovary Syndrome: Is the Evidence Enough? | Abstract/Protocol/Opinion/Review |  |
| 2008 | Hoeger, K. M. | Exercise therapy in polycystic ovary syndrome | Abstract/Protocol/Opinion/Review |  |
| 2021 | Morris, Alan | Exercise training in women with PCOS - finding clarity | Abstract/Protocol/Opinion/Review |  |
| 2021 | Morris, A. | Exercise training in women with PCOS — finding clarity | Abstract/Protocol/Opinion/Review |  |
| 2011 | Roessler, K. K.-//-Andersen, M.-//-Glintborg, D.-//-Ravn, P.-//-Birkebaek, C. | Group counselling and physical activity of patients with polycystic ovarial syndrome (PCOS) | Abstract/Protocol/Opinion/Review |  |
| 2021 | Fong, S. L.-//-Douma, A.-//-Verhaeghe, J. | Implementing the international evidence-based guideline of assessment and management of polycystic ovary syndrome (PCOS): how to achieve weight loss in overweight and obese women with PCOS? | Abstract/Protocol/Opinion/Review |  |
| 2022 | Gu, Y.-//-Zhou, G.-//-Zhou, F.-//-Wu, Q.-//-Ma, C.-//-Zhang, Y.-//-Ding, J.-//-Hua, K. | Life Modifications and PCOS: Old Story But New Tales | Abstract/Protocol/Opinion/Review |  |
| 2019 | Lim, S. S.-//-Hutchison, S. K.-//-Van Ryswyk, E.-//-Norman, R. J.-//-Teede, H. J.-//-Moran, L. J. | Lifestyle changes in women with polycystic ovary syndrome | Abstract/Protocol/Opinion/Review |  |
| 2006 | Norman, R. J.-//-Homan, G.-//-Moran, L.-//-Noakes, M. | Lifestyle choices, diet, and insulin sensitizers in polycystic ovary syndrome | Abstract/Protocol/Opinion/Review |  |
| 2023 | Cowan, S.-//-Lim, S.-//-Alycia, C.-//-Pirotta, S.-//-Thomson, R.-//-Gibson-Helm, M.-//-Blackmore, R.-//-Naderpoor, N.-//-Bennett, C.-//-Ee, C.-//-Rao, V. B. T.-//-Mousa, A.-//-Alesi, S.-//-Moran, L. | Lifestyle management in polycystic ovary syndrome - beyond diet and physical activity | Abstract/Protocol/Opinion/Review |  |
| 2023 | Cowan, Stephanie-//-Lim, Siew-//-Alycia, Chelsea-//-Pirotta, Stephanie-//-Thomson, Rebecca-//-Gibson-Helm, Melanie-//-Blackmore, Rebecca-//-Naderpoor, Negar-//-Bennett, Christie-//-Ee, Carolyn-//-Rao, Vibhuti-//-Mousa, Aya-//-Alesi, Simon-//-Moran, Lisa | Lifestyle management in polycystic ovary syndrome – beyond diet and physical activity | Abstract/Protocol/Opinion/Review |  |
| 2007 | Yasmin, E.-//-Balen, A. H. | Management of polycystic ovary syndrome | Abstract/Protocol/Opinion/Review |  |
| 2001 | Norman, R. J.-//-Kidson, W. J.-//-Cuneo, R. C.-//-Zacharin, M. R.-//-Endocrine Soc, Australia-//-Australian Diabet, Soc-//-Australasian Paediat Endocrine, Grp | Metformin and intervention in polycystic ovary syndrome | Abstract/Protocol/Opinion/Review |  |
| 2013 | Ravn, P.-//-Haugen, A. G.-//-Glintborg, D. | Overweight in polycystic ovary syndrome. An update on evidence based advice on diet, exercise and metformin use for weight loss | Abstract/Protocol/Opinion/Review |  |
| 2016 | Yildiz, B. O. | Reproductive endocrinology: Contraceptives, exercise and diet-are all three needed in PCOS? | Abstract/Protocol/Opinion/Review |  |
| 2021 | Wright, P. J.-//-Corbett, C. F.-//-Pinto, B. M.-//-Dawson, R. M.-//-Wirth, M. | Resistance Training as Therapeutic Management in Women with PCOS: What is the Evidence? | Abstract/Protocol/Opinion/Review |  |
| 2020 | Woodward, A.-//-Broom, D.-//-Dalton, C.-//-Metwally, M.-//-Klonizakis, M. | Supervised exercise training and increased physical activity to reduce cardiovascular disease risk in women with polycystic ovary syndrome: study protocol for a randomized controlled feasibility trial | Abstract/Protocol/Opinion/Review |  |
| 2019 | Hiam, D.-//-Patten, R.-//-Gibson-Helm, M.-//-Moreno-Asso, A.-//-McIlvenna, L.-//-Levinger, I.-//-Harrison, C.-//-Moran, L. J.-//-Joham, A.-//-Parker, A.-//-Shorakae, S.-//-Simar, D.-//-Stepto, N. | The effectiveness of high intensity intermittent training on metabolic, reproductive and mental health in women with polycystic ovary syndrome: study protocol for the iHIT- randomised controlled trial | Abstract/Protocol/Opinion/Review |  |
| 2020 | Khalafi, M.-//-Symonds, M. E. | The impact of high-intensity interval training on inflammatory markers in metabolic disorders: A meta-analysis | Abstract/Protocol/Opinion/Review |  |
| 2020 | Khalafi, Mousa-//-Symonds, Michael E. | The impact of high‐intensity interval training on inflammatory markers in metabolic disorders: A meta‐analysis | Abstract/Protocol/Opinion/Review |  |
| 2018 | Lara, L.-//-Lopes, I. P.-//-Dos Reis, R. M.-//-Ribeiro, V. B.-//-De Souza, H. C. D.-//-Silva, R. C. | Aerobic physical training improves sexual function and qol of pcos women: Randomized conrrolled tria | Abstract/Protocol/Opinion/Review |  |
| 2020 | Tabassum, F.-//-Sinha, H. H.-//-Dhar, K.-//-Jyoti, C.-//-Akhtar, M. S.-//-Chopra, V. S. | Assessment of Psycho-emotional Distress Due to Age, Body Mass Index, and Marital Status in Polycystic Ovary Syndrome in North Indian Population | Different intervention |  |
| 2021 | Mizgier, M.-//-Watrowski, R.-//-Opydo-Szymaczek, J.-//-Jodłowska-Siewert, E.-//-Lombardi, G.-//-Kędzia, W.-//-Jarząbek-Bielecka, G. | Association of Macronutrients Composition, Physical Activity and Serum Androgen Concentration in Young Women with Polycystic Ovary Syndrome | Different intervention |  |
| 2021 | Parveen, N.-//-Alanizy, A. M.-//-Alrowiliy, G. F.-//-Alshammari, H. O. | Awareness of polycystic ovarian syndrome and effect of lifestyle modification on its management among female medical students at Hail University | Different intervention |  |
| 2023 | Kite, C.-//-Atkinson, L.-//-McGregor, G.-//-Clark, C. C. T.-//-Randeva, H. S.-//-Kyrou, I. | Capability, Opportunity, and Motivation-Identifying Constructs for Increasing Physical Activity Behaviours in Women with Polycystic Ovary Syndrome (PCOS) | Different intervention |  |
| 2010 | Fux Otta, C.-//-Wior, M.-//-Iraci, G. S.-//-Kaplan, R.-//-Torres, D.-//-Gaido, M. I.-//-Wyse, E. P. | Clinical, metabolic, and endocrine parameters in response to metformin and lifestyle intervention in women with polycystic ovary syndrome: a randomized, double-blind, and placebo control trial | Different intervention |  |
| 2004 | Wright, C. E.-//-Zborowski, J. V.-//-Talbott, E. O.-//-McHugh-Pemu, K.-//-Youk, A. | Dietary intake, physical activity, and obesity in women with polycystic ovary syndrome | Different intervention |  |
| 2016 | El-Bandrawy, A. M.-//-Ghareeb, H. O. | Effect of laser puncture combined with a diet-exercise intervention on obese polycystic ovarian females | Different intervention |  |
| 2022 | Guo, Y.-//-Liu, Y.-//-Yan, X.-//-Ding, R.-//-Wang, L. | Effects of multidimensional life management on healthy behavior in polycystic ovary syndrome patients: A randomized controlled trial | Different intervention |  |
| 2020 | Benrick, A.-//-Pillon, N. J.-//-Nilsson, E.-//-Lindgren, E.-//-Krook, A.-//-Ling, C.-//-Stener-Victorin, E. | Electroacupuncture mimics exercise-induced changes in skeletal muscle gene expression in women with polycystic ovary syndrome | Different intervention |  |
| 2023 | Cowan, S.-//-Grassi, A.-//-Monahan Couch, L.-//-Jeanes, Y.-//-Lim, S.-//-Pirotta, S.-//-Harris, J.-//-McGirr, C.-//-Moran, L. | Evidence-Based Lifestyle Guidelines and Self-Management Strategies Utilized by Women with Polycystic Ovary Syndrome | Different intervention |  |
| 2018 | Huang, D.-//-Greenwood, E.-//-Kao, C.-//-Quinn, M.-//-Cedars, M.-//-Huddleston, H. | EXERCISE BEHAVIORS BY ETHNIC GROUP AMONG PATIENTS WITH POLYCYSTIC OVARY SYNDROME | Different intervention |  |
| 2002 | Randeva, H. S.-//-Lewandowski, K. C.-//-Drzewoski, J.-//-Brooke-Wavell, K.-//-O'Callaghan, C.-//-Czupryniak, L.-//-Hillhouse, E. W.-//-Prelevic, G. M. | Exercise decreases plasma total homocysteine in overweight young women with polycystic ovary syndrome | Different intervention |  |
| 2016 | Shishehgar, F.-//-Tehrani, F. R.-//-Mirmiran, P.-//-Hajian, S.-//-Baghestani, A. R.-//-Moslehi, N. | Factors Influencing Physical Activity in Women with Polycystic Ovary Syndrome in Comparison to Eumenorrheic Non Hirsute Women | Different intervention |  |
| 2018 | Ee, C.-//-Smith, C.-//-Costello, M.-//-MacMillan, F.-//-Moran, L.-//-Baylock, B.-//-Teede, H. | Feasibility and acceptability of a proposed trial of acupuncture as an adjunct to lifestyle interventions for weight loss in Polycystic Ovary Syndrome: a qualitative study | Different intervention |  |
| 2011 | Barr, S.-//-Hart, K.-//-Reeves, S.-//-Sharp, K.-//-Jeanes, Y. M. | Habitual dietary intake, eating pattern and physical activity of women with polycystic ovary syndrome | Different intervention |  |
| 2020 | Copp, T.-//-Cvejic, E.-//-McCaffery, K.-//-Hersch, J.-//-Doust, J.-//-Mol, B. W.-//-Dokras, A.-//-Mishra, G.-//-Jansen, J. | Impact of a diagnosis of polycystic ovary syndrome on diet, physical activity and contraceptive use in young women: findings from the Australian Longitudinal Study of Women's Health | Different intervention |  |
| 2010 | Harris-Glocker, M.-//-Davidson, K.-//-Kochman, L.-//-Guzick, D.-//-Hoeger, K. | Improvement in quality-of-life questionnaire measures in obese adolescent females with polycystic ovary syndrome treated with lifestyle changes and oral contraceptives, with or without metformin | Different intervention |  |
| 2021 | Pirotta, S.-//-Joham, A.-//-Moran, L.-//-Lim, S.-//-Skouteris, H. | Informing the design and delivery of a lifestyle program for women with polycystic ovary syndrome: A mixed-methods investigation on patients' perspectives | Different intervention |  |
| 2021 | Awoke, M. A.-//-Earnest, A.-//-Joham, A.-//-Hodge, A.-//-Brown, W.-//-Teede, H.-//-Moran, L. | Longitudinal weight gain and lifestyle factors in women with and without polycystic ovary syndrome | Different intervention |  |
| 2013 | Rupa Vani, K.-//-Veena, K. S.-//-Subitha, L.-//-Hemanth Kumar, V. R.-//-Bupathy, A. | Menstrual abnormalities in school going girls - Are they related to dietary and exercise pattern? | Different intervention |  |
| 2017 | Greenwood, E. A.-//-Kao, C. N.-//-Cedars, M.-//-Huddleston, H. G. | On your feet: Is sitting time linked to adverse metabolic profiles in polycystic ovary syndrome, independent of exercise? | Different intervention |  |
| 2022 | Young, C. C.-//-Monge, M.-//-Minami, H.-//-Rew, L.-//-Conroy, H.-//-Peretz, C.-//-Tan, L. | Outcomes of a Mindfulness-Based Healthy Lifestyle Intervention for Adolescents and Young Adults with Polycystic Ovary Syndrome | Different intervention |  |
| 2014 | Banting, L. K.-//-Gibson-Helm, M.-//-Polman, R.-//-Teede, H. J.-//-Stepto, N. K. | Physical activity and mental health in women with Polycystic Ovary Syndrome | Different intervention |  |
| 2011 | Lamb, J. D.-//-Johnstone, E. B.-//-Rousseau, J. A.-//-Jones, C. L.-//-Pasch, L. A.-//-Cedars, M. I.-//-Huddleston, H. G. | Physical activity in women with polycystic ovary syndrome: prevalence, predictors, and positive health associations | Different intervention |  |
| 2021 | Manteghi, G.-//-Shahraki, Z.-//-Moghadam, M. N.-//-Ghanbarpour, M. H. | Pregnancy outcome in PCOS patients: The effects of letrozol combined with exercise | Different intervention |  |
| 2010 | Palomba, S.-//-Falbo, A.-//-Giallauria, F.-//-Russo, T.-//-Rocca, M.-//-Tolino, A.-//-Zullo, F.-//-Orio, F. | Six weeks of structured exercise training and hypocaloric diet increases the probability of ovulation after clomiphene citrate in overweight and obese patients with polycystic ovary syndrome: a randomized controlled trial | Different intervention |  |
| 2020 | Chen, H.-//-Wang, Q. | The effects of lifestyle guidance and mental health care on improving the carbohydrate metabolism and enhancing the pregnancy rate in obese pcos patients mental health care | Different intervention |  |
| 2012 | Wang, Q. Y.-//-Huang, W.-//-Song, Y. S.-//-Li, X.-//-Shen, L. L. | The impact of oral contraceptives, metformin and lifestyle modification on the metabolism disorder in polycystic ovary syndrome women: A randomized controlled trial | Different intervention |  |
| 2014 | Panidis, D.-//-Tziomalos, K.-//-Papadakis, E.-//-Chatzis, P.-//-Kandaraki, E. A.-//-Tsourdi, E. A.-//-Katsikis, I. | The role of orlistat combined with lifestyle changes in the management of overweight and obese patients with polycystic ovary syndrome | Different intervention |  |
| 2017 | Zhang, J.-//-Si, Q.-//-Li, J. | Therapeutic effects of metformin and clomiphene in combination with lifestyle intervention on infertility in women with obese polycystic ovary syndrome | Different intervention |  |
| 2021 | Awoke, M. A.-//-Earnest, A.-//-Joham, A. E.-//-Hodge, A. M.-//-Teede, H. J.-//-Brown, W. J.-//-Moran, L. J. | Weight gain and lifestyle factors in women with and without polycystic ovary syndrome | Different intervention |  |
| 2017 | Moran, L. J.-//-Brown, W. J.-//-McNaughton, S. A.-//-Joham, A. E.-//-Teede, H. J. | Weight management practices associated with PCOS and their relationships with diet and physical activity | Different intervention |  |
| 2020 | Jiskoot, G.-//-Timman, R.-//-Beerthuizen, A.-//-Dietz de Loos, A.-//-Busschbach, J.-//-Laven, J. | Weight Reduction Through a Cognitive Behavioral Therapy Lifestyle Intervention in PCOS: The Primary Outcome of a Randomized Controlled Trial | Different intervention |  |
| 2009 | Maurer, M. M.-//-Burkhoff, D.-//-Maybaum, S.-//-Franco, V.-//-Vittorio, T. J.-//-Williams, P.-//-White, L.-//-Kamalakkannan, G.-//-Myers, J.-//-Mancini, D. M.-//-Maurer, Mathew M.-//-Burkhoff, Daniel-//-Maybaum, Simon-//-Franco, Veronica-//-Vittorio, Timothy J.-//-Williams, Paula-//-White, Leah-//-Kamalakkannan, Gayathri-//-Myers, Jonathan-//-Mancini, Donna M. | A multicenter study of noninvasive cardiac output by bioreactance during symptom-limited exercise | Different population |  |
| 2017 | Parker, L.-//-Shaw, C. S.-//-Banting, L.-//-Levinger, I.-//-Hill, K. M.-//-McAinch, A. J.-//-Stepto, N. K. | Acute Low-Volume High-Intensity Interval Exercise and Continuous Moderate Intensity Exercise Elicita Similar Improvement in 24-h Glycemic Control in Overweight and Obese Adults | Different population |  |
| 2006 | Vega, S. R.-//-Strüder, H. K.-//-Wahrmann, B. V.-//-Bloch, W.-//-Hollmann, W. | Bicarbonate reduces serum prolactin increase induced by exercise to exhaustion | Different population |  |
| 2018 | Yang, H. | Can gestational diabetes be prevented (the role of exercise, diet, and changes in life style) | Different population |  |
| 2016 | Rodino, I. S.-//-Byrne, S.-//-Sanders, K. A. | Disordered eating attitudes and exercise in women undergoing fertility treatment | Different population |  |
| 2018 | Gorkem, Umit-//-Yamaner, Faruk-//-Demirkan, Erkan-//-Inal, Hasan Ali | Does the spinning exercise affect the ovarian reserve in reproductive-young women? | Different population |  |
| 2002 | Somfay, Attila-//-Pórszász, János-//-Sang-Moo, Lee-//-Casaburi, Richard-//-Pórszász, János-//-Lee, Sang-Moo | Effect of hyperoxia on gas exchange and lactate kinetics following exercise onset in nonhypoxemic COPD patients | Different population |  |
| 2005 | Wing-Gaia, S. L.-//-Subudhi, A. W.-//-Askew, E. W. | Effects of purified oxygenated water on exercise performance during acute hypoxic exposure | Different population |  |
| 2019 | Nithyanisha, R.-//-Ashwini, S.-//-Mary, S. M. D.-//-Kirupa, K.-//-Lochani, V. P. | Efficacy of aerobic exercise and relaxation training in premenstrual symptoms in collegiates | Different population |  |
| 2015 | Pedersen, B. K.-//-Saltin, B. | Exercise as medicine - evidence for prescribing exercise as therapy in 26 different chronic diseases | Different population |  |
| 2006 | Kaaja, R. J.-//-Pöyhönen-Alho, M. K. | Insulin resistance and sympathetic overactivity in women | Different population |  |
| 2004 | Hawley, J. A.-//-Houmard, J. A. | Introduction - preventing insulin resistance through exercise: a cellular approach | Different population |  |
| 2012 | Booth, F. W.-//-Roberts, C. K.-//-Laye, M. J. | Lack of exercise is a major cause of chronic diseases | Different population |  |
| 2007 | Hansen, J. E.-//-Ulubay, G.-//-Chow, B. F.-//-Sun, X. G.-//-Wasserman, K. | Mixed-expired and end-tidal CO2 distinguish between ventilation and perfusion defects during exercise testing in patients with lung and heart diseases | Different population |  |
| 2013 | Roman, Michael-//-Casaburi, James-//-Porszasz, Janos-//-Casaburi, Richard | Noninvasive assessment of normality of V/ V in clinical cardiopulmonary exercise testing utilizing incremental cycle ergometry | Different population |  |
| 2015 | Çakır-Atabek, H.-//-Özdemir, F.-//-Çolak, R. | Oxidative stress and antioxidant responses to progressive resistance exercise intensity in trained and untrained males | Different population |  |
| 2019 | Joshi, A.-//-Shinde, R.-//-Page, A.-//-Shinde, S. | Oxygen uptake responses (Vo2 Max) in type 2 diabetes mellitus (T2DM) and healthy individuals using cardiopulmonary exercise testing | Different population |  |
| 2012 | Vega, Sandra Rojas-//-Hollmann, W.-//-Wahrmann, B. Vera-//-StrÃ¼der, H. K. | pH Buffering Does not Influence BDNF Responses to Exercise | Different population |  |
| 2009 | Vilke, G. M.-//-Sloane, C. M.-//-Suffecool, A.-//-Kolkhorst, F. W.-//-Neuman, T. S.-//-Castillo, E. M.-//-Chan, T. C. | Physiologic effects of the TASER after exercise | Different population |  |
| 2016 | Green, D. J.-//-Hopkins, N. D.-//-Jones, H.-//-Thijssen, D. H. J.-//-Eijsvogels, T. M. H.-//-Yeap, B. B. | Sex differences in vascular endothelial function and health in humans: impacts of exercise | Different population |  |
| 2001 | Tanabe, Y.-//-Hosaka, Y.-//-Ito, M.-//-Ito, E.-//-Suzuki, K.-//-Tanabe, Y.-//-Hosaka, Y.-//-Ito, M.-//-Ito, E.-//-Suzuki, K. | Significance of end-tidal P(CO(2)) response to exercise and its relation to functional capacity in patients with chronic heart failure | Different population |  |
| 2011 | Olfert, I. M.-//-Loeckinger, A.-//-Treml, B.-//-Faulhaber, M.-//-Flatz, M.-//-Burtscher, M.-//-Truebsbach, S.-//-Kleinsasser, A.-//-Olfert, I. Mark-//-Loeckinger, Alexander-//-Treml, Benedikt-//-Faulhaber, Martin-//-Flatz, Markus-//-Burtscher, Martin-//-Truebsbach, Susanne-//-Kleinsasser, Axel | Sildenafil and bosentan improve arterial oxygenation during acute hypoxic exercise: a controlled laboratory trial | Different population |  |
| 2014 | Christiansen, S. C.-//-Vanky, E.-//-Klungland, H.-//-Stafne, S. N.-//-Mørkved, S.-//-Salvesen, KÅ-//-Sæther, M.-//-Carlsen, S. M. | The effect of exercise and metformin treatment on circulating free DNA in pregnancy | Different population |  |
| 2018 | Kazemi, M.-//-McBreairty, L. E.-//-Chizen, D. R.-//-Pierson, R. A.-//-Chilibeck, P. D.-//-Zello, G. A. | A Comparison of a Pulse-Based Diet and the Therapeutic Lifestyle Changes Diet in Combination with Exercise and Health Counselling on the Cardio-Metabolic Risk Profile in Women with Polycystic Ovary Syndrome: A Randomized Controlled Trial | Different study design |  |
| 2015 | Miranda-Furtado, C. L.-//-Ramos, F. K. P.-//-Kogure, G. S.-//-Santana-Lemos, B. A.-//-Ferriani, R. A.-//-Calado, R. T.-//-Dos Reis, R. M. | A Nonrandomized Trial of Progressive Resistance Training Intervention in Women with Polycystic Ovary Syndrome and Its Implications in Telomere Content | Different study design |  |
| 2022 | Dashti, S.-//-Abdul Hamid, H.-//-Mohamad Saini, S.-//-Tusimin, M.-//-Ismail, M.-//-Jafarzadeh Esfehani, A.-//-Ching, S. M.-//-Lee, K. W.-//-Ismail, N.-//-Wong, J. L.-//-Abdul Latiff, L. | A randomised controlled trial on the effects of a structural education module among women with polycystic ovarian syndrome on nutrition and physical activity changes | Different study design |  |
| 2017 | McBreairty, L. E.-//-Kazemi, M.-//-Gordon, J. J.-//-Pierson, R. A.-//-Chizen, D. R.-//-Chilibeck, P. D.-//-Zello, G. A. | A Randomized Clinical Trial in Women with Polycystic Ovary Syndrome: Effects of a Pulse-Based Diet and Exercise Intervention on Blood Lipids, Body Composition and Reproductive Measures | Different study design |  |
| 2020 | Kazemi, M.-//-Pierson, R. A.-//-McBreairty, L. E.-//-Chilibeck, P. D.-//-Zello, G. A.-//-Chizen, D. R. | A randomized controlled trial of a lifestyle intervention with longitudinal follow-up on ovarian dysmorphology in women with polycystic ovary syndrome | Different study design |  |
| 2008 | Giallauria, F.-//-Palomba, S.-//-Manguso, F.-//-Vitelli, A.-//-Maresca, L.-//-Tafuri, D.-//-Lombardi, G.-//-Colao, A.-//-Vigorito, C.-//-Orio, F. | Abnormal heart rate recovery after maximal cardiopulmonary exercise stress testing in young overweight women with polycystic ovary syndrome | Different study design |  |
| 2021 | Lionett, S.-//-Kiel, I. A.-//-Røsbjørgen, R.-//-Lydersen, S.-//-Larsen, S.-//-Moholdt, T. | Absent Exercise-Induced Improvements in Fat Oxidation in Women With Polycystic Ovary Syndrome After High-Intensity Interval Training | Different study design |  |
| 2017 | Dantas, W. S.-//-Murai, I. H.-//-Perandini, L. A.-//-Azevedo, H.-//-Moreira-Filho, C. A.-//-Camara, N. O.-//-Roschel, H.-//-Gualano, B. | Acute exercise elicits differential expression of insulin resistance genes in the skeletal muscle of patients with polycystic ovary syndrome | Different study design |  |
| 2016 | Sá, J. C.-//-Costa, E. C.-//-da Silva, E.-//-Tamburús, N. Y.-//-Porta, A.-//-Medeiros, L. F.-//-Lemos, T. M.-//-Soares, E. M.-//-Azevedo, G. D. | Aerobic exercise improves cardiac autonomic modulation in women with polycystic ovary syndrome | Different study design |  |
| 2013 | Costa, E. C.-//-De Sá, J. C. F.-//-De Medeiros, R. D.-//-Soares, E. M. M.-//-Azevedo, G. D. | Aerobic exercise improves ovarian morphology of women with polycystic ovary syndrome and is perceived as a pleasurable intervention | Different study design |  |
| 2011 | Redman, L. M.-//-Elkind-Hirsch, K.-//-Ravussin, E. | Aerobic exercise in women with polycystic ovary syndrome improves ovarian morphology independent of changes in body composition | Different study design |  |
| 2009 | Moro, C.-//-Pasarica, M.-//-Elkind-Hirsch, K.-//-Redman, L. M. | Aerobic exercise training improves atrial natriuretic peptide and catecholamine-mediated lipolysis in obese women with polycystic ovary syndrome | Different study design |  |
| 2022 | Souza, Hcdd-//-Philbois, S. V.-//-Facioli, T. D.-//-Ferriani, R. A.-//-Gastaldi, A. C. | Aerobic physical training impact on adipokines in women with polycystic ovary syndrome-Effects of body fat percentage | Different study design |  |
| 2018 | Miranda-Furtado, C. L.-//-Ribeiro, V. B.-//-Lopes, I. P.-//-Silva, R. C.-//-Kogure, G. S.-//-Pedroso, D. C.-//-Ferriani, R. A.-//-Reis, R. M. | AEROBIC PHYSICAL TRAINING REDUCES ANTHROPOMETRIC INDEXES AND HYPERANDROGENISM IN POLYCYSTIC OVARY SYNDROME | Different study design |  |
| 2016 | Rissanen, A. P.-//-Koskela-Koivisto, T.-//-Hägglund, H.-//-Koponen, A. S.-//-Aho, J. M.-//-Pöyhönen-Alho, M.-//-Tiitinen, A.-//-Tikkanen, H. O.-//-Peltonen, J. E. | Altered cardiorespiratory response to exercise in overweight and obese women with polycystic ovary syndrome | Different study design |  |
| 2007 | Vigorito, C.-//-Giallauria, F.-//-Palomba, S.-//-Cascella, T.-//-Manguso, F.-//-Lucci, R.-//-De Lorenzo, A.-//-Tafuri, D.-//-Lombardi, G.-//-Colao, A.-//-Orio, F. | Beneficial effects of a three-month structured exercise training program on cardiopulmonary functional capacity in young women with polycystic ovary syndrome | Different study design |  |
| 2022 | Kumar, M. Prem-//-Preethi Angela, S. Jeya-//-Kavitha, S. | Benefit of Stability Exercise on Swiss Ball Exercise along with Treadmill Walking in Physiotherapy College Girl Students with PCOS Symptoms: Single Group Pre Post Design | Different study design |  |
| 2015 | Turan, V.-//-Mutlu, E. K.-//-Solmaz, U.-//-Ekin, A.-//-Tosun, O.-//-Tosun, G.-//-Mat, E.-//-Gezer, C.-//-Malkoc, M. | Benefits of short-term structured exercise in non-overweight women with polycystic ovary syndrome: a prospective randomized controlled study | Different study design |  |
| 2022 | Philbois, S. V.-//-Ribeiro, V. B.-//-Tank, J.-//-Dos Reis, R. M.-//-Gerlach, D. A.-//-Souza, H. C. D. | Cardiovascular autonomic modulation differences between moderate-intensity continuous and high-intensity interval aerobic training in women with PCOS: A randomized trial | Different study design |  |
| 2022 | Kiel, I. A.-//-Jones, H.-//-Lionett, S.-//-Røsbjørgen, R.-//-Lydersen, S.-//-Vanky, E.-//-Moholdt, T. | Cardiovascular Health Does Not Change Following High-Intensity Interval Training in Women with Polycystic Ovary Syndrome | Different study design |  |
| 2020 | Lionett, S.-//-Kiel, I. A.-//-Camera, D. M.-//-Vanky, E.-//-Parr, E. B.-//-Lydersen, S.-//-Hawley, J. A.-//-Moholdt, T. | Circulating and Adipose Tissue miRNAs in Women With Polycystic Ovary Syndrome and Responses to High-Intensity Interval Training | Different study design |  |
| 2019 | Kirk, R. J.-//-Madden, L. A.-//-Peart, D. J.-//-Aye, M. M.-//-Atkin, S. L.-//-Vince, R. V. | Circulating Endothelial Microparticles Reduce in Concentration Following an Exercise Programme in Women With Polycystic Ovary Syndrome | Different study design |  |
| 2023 | Zhou, X.-//-Wang, Y.-//-Chen, W.-//-Zhang, H.-//-He, Y.-//-Dai, H.-//-Hu, W.-//-Li, K.-//-Zhang, L.-//-Chen, C.-//-Yang, G.-//-Li, L. | Circulating HHIP Levels in Women with Insulin Resistance and PCOS: Effects of Physical Activity, Cold Stimulation and Anti-Diabetic Drug Therapy | Different study design |  |
| 2021 | Benham, J. L.-//-Booth, J. E.-//-Friedenreich, C. M.-//-Rabi, D. M.-//-Sigal, R. J. | Comparative Success of Recruitment Strategies for an Exercise Intervention Trial Among Women With Polycystic Ovary Syndrome: Observational Study | Different study design |  |
| 2009 | Thomson, R. L.-//-Buckley, J. D.-//-Moran, L. J.-//-Noakes, M.-//-Clifton, P. M.-//-Norman, R. J.-//-Brinkworth, G. D. | Comparison of aerobic exercise capacity and muscle strength in overweight women with and without polycystic ovary syndrome | Different study design |  |
| 2016 | Wang, Q. Y.-//-Song, Y.-//-Huang, W.-//-Xiao, L.-//-Wang, Q. S.-//-Feng, G. M. | Comparison of Drospirenone- with Cyproterone Acetate-Containing Oral Contraceptives, Combined with Metformin and Lifestyle Modifications in Women with Polycystic Ovary Syndrome and Metabolic Disorders: A Prospective Randomized Control Trial | Different study design |  |
| 2020 | Furtado, C. L. M.-//-Barbosa Ribeiro, V.-//-Pedroso, D. C.-//-Kogure, G. S.-//-Ferriani, R. A.-//-Calado, R. T.-//-dos Reis, R. M. | CONTINUOUS AND INTERMITTENT AEROBIC TRAINING DID NOT CHANGE TELOMERE LENGTH, ALTHOUGH IT REDUCES HYPERANDROGENISM AND ANTROPOMETRIC INDEXES IN POLYCYSTIC OVARY SYNDROME | Different study design |  |
| 2011 | Álvarez-Blasco, F.-//-Luque-Ramírez, M.-//-Escobar-Morreale, H. F. | Diet composition and physical activity in overweight and obese premenopausal women with or without polycystic ovary syndrome | Different study design |  |
| 2013 | Vosnakis, C.-//-Georgopoulos, N. A.-//-Rousso, D.-//-Mavromatidis, G.-//-Katsikis, I.-//-Roupas, N. D.-//-Mamali, I.-//-Panidis, D. | Diet, physical exercise and Orlistat administration increase serum anti-Müllerian hormone (AMH) levels in women with polycystic ovary syndrome (PCOS) | Different study design |  |
| 2019 | Lin, A. W.-//-Kazemi, M.-//-Jarrett, B. Y.-//-Vanden Brink, H.-//-Hoeger, K. M.-//-Spandorfer, S. D.-//-Lujan, M. E. | Dietary and Physical Activity Behaviors in Women with Polycystic Ovary Syndrome per the New International Evidence-Based Guideline | Different study design |  |
| 2021 | Mizgier, M.-//-Jarzabek-Bielecka, G.-//-Formanowicz, D.-//-Jodlowska-Siewert, E.-//-Mruczyk, K.-//-Cisek-Wozniak, A.-//-Kedzia, W.-//-Opydo-Szymaczek, J. | Dietary and Physical Activity Habits in Adolescent Girls with Polycystic Ovary Syndrome (PCOS)-HAstudy | Different study design |  |
| 2021 | Wang, Z.-//-Groen, H.-//-Cantineau, A. E. P.-//-van Elten, T. M.-//-Karsten, M. D. A.-//-van Oers, A. M.-//-Mol, B. W. J.-//-Roseboom, T. J.-//-Hoek, A. | Dietary Intake, Eating Behavior, Physical Activity, and Quality of Life in Infertile Women with PCOS and Obesity Compared with Non-PCOS Obese Controls | Different study design |  |
| 2018 | Aye, M. M.-//-Butler, A. E.-//-Kilpatrick, E. S.-//-Kirk, R.-//-Vince, R.-//-Rigby, A. S.-//-Sandeman, D.-//-Atkin, S. L. | Dynamic Change in Insulin Resistance Induced by Free Fatty Acids Is Unchanged Though Insulin Sensitivity Improves Following Endurance Exercise in PCOS | Different study design |  |
| 2019 | Faryadian, B.-//-Tadibi, V.-//-Behpour, N. | Effect of 12-week High Intensity Interval Training Program on C-Reactive Protein and Insulin Resistance in Women with Polycystic Ovary Syndrome | Different study design |  |
| 2019 | Veena Kirthika, S.-//-Paul, J.-//-Senthil Selvam, P.-//-Sathya Priya, V. | Effect of aerobic exercise and life style intervention among young women with polycystic ovary syndrome | Different study design |  |
| 2022 | Elbandrawy, A. M.-//-Yousef, A. M.-//-Morgan, E. N.-//-Ewais, N. F.-//-Eid, M. M.-//-Elkholi, S. M.-//-Abdelbasset, W. K. | Effect of aerobic exercise on inflammatory markers in polycystic ovary syndrome: a randomized controlled trial | Different study design |  |
| 2019 | Parseh, S.-//-Shakerian, S.-//-Alizadeh, A. A. | Effect of Chronic Aerobic/Resistive Exercises with Supplementation of Cinnamon on Insulin Resistance in Women with Polycystic Ovary Syndrome in Ahvaz City in 2017 | Different study design |  |
| 2019 | Gilani, N.-//-Rad, F. Z.-//-Ebrahimi, M.-//-Haghshenas, R. | Effect of Eight Weeks Endurance Training on Ovarian Androgens in Women with Polycystic Ovary Syndrome: Application of Multivariate Longitudinal Models | Different study design |  |
| 2021 | Cochrane, T.-//-Tengku-Kamalden, T. F.-//-Davey, R.-//-Dev, R. D. O. | Effect of Exercise and Weight Loss in Polycystic Ovarian Syndrome among Obese Women | Different study design |  |
| 2012 | Hutchison, S. K.-//-Teede, H. J.-//-Rachoń, D.-//-Harrison, C. L.-//-Strauss, B. J.-//-Stepto, N. K. | Effect of exercise training on insulin sensitivity, mitochondria and computed tomography muscle attenuation in overweight women with and without polycystic ovary syndrome | Different study design |  |
| 2011 | Lass, N.-//-Kleber, M.-//-Winkel, K.-//-Wunsch, R.-//-Reinehr, T. | Effect of lifestyle intervention on features of polycystic ovarian syndrome, metabolic syndrome, and intima-media thickness in obese adolescent girls | Different study design |  |
| 2021 | Ramanjaneya, M.-//-Abdalhakam, I.-//-Bettahi, I.-//-Bensila, M.-//-Jerobin, J.-//-Aye, M. M.-//-Alkasem, M.-//-Sathyapalan, T.-//-Atkin, S. L.-//-Abou-Samra, A. B. | Effect of Moderate Aerobic Exercise on Complement Activation Pathways in Polycystic Ovary Syndrome Women | Different study design |  |
| 2019 | Esmael, M. E. H.-//-Abdelsamea, G. A.-//-Nashed, A. B. | Effect of pulsed electromagnetic field versus aerobic exercises on women with polycystic ovary syndrome: A single-blind randomized controlled trial | Different study design |  |
| 2021 | Wang, Z.-//-Groen, H.-//-Cantineau, A. E. P.-//-van Elten, T. M.-//-Karsten, M. D. A.-//-van Oers, A. M.-//-Mol, B. W. J.-//-Roseboom, T. J.-//-Hoek, A. | Effectiveness of a 6-Month Lifestyle Intervention on Diet, Physical Activity, Quality of Life, and Markers of Cardiometabolic Health in Women with PCOS and Obesity and Non-PCOS Obese Controls: One Size Fits All? | Different study design |  |
| 2013 | Nidhi, R.-//-Padmalatha, V.-//-Nagarathna, R.-//-Amritanshu, R. | Effects of a holistic yoga program on endocrine parameters in adolescents with polycystic ovarian syndrome: a randomized controlled trial | Different study design |  |
| 2015 | Abazar, E.-//-Taghian, F.-//-Mardanian, F.-//-Forozandeh, D. | Effects of aerobic exercise on plasma lipoproteins in overweight and obese women with polycystic ovary syndrome | Different study design |  |
| 2020 | Ribeiro, V. B.-//-Kogure, G. S.-//-Lopes, I. P.-//-Silva, R. C.-//-Pedroso, D. C. C.-//-de Melo, A. S.-//-de Souza, H. C. D.-//-Ferriani, R. A.-//-Miranda Furtado, C. L.-//-Dos Reis, R. M. | Effects of continuous and intermittent aerobic physical training on hormonal and metabolic profile, and body composition in women with polycystic ovary syndrome: A randomized controlled trial | Different study design |  |
| 2018 | Liu, Ying-//-Liu, Xiaofang-//-Zhang, Jing-//-Zhou, Kunyan-//-Luo, Li-//-Xu, Liangzhi | Effects of exercise and dietary habits on the occurrence of polycystic ovary syndrome over 5 years of follow-up | Different study design |  |
| 2013 | Roessler, K. K.-//-Birkebaek, C.-//-Ravn, P.-//-Andersen, M. S.-//-Glintborg, D. | Effects of exercise and group counselling on body composition and VO2max in overweight women with polycystic ovary syndrome | Different study design |  |
| 2006 | Bruner, B.-//-Chad, K.-//-Chizen, D. | Effects of exercise and nutritional counseling in women with polycystic ovary syndrome | Different study design |  |
| 2011 | Hutchison, S. K.-//-Stepto, N. K.-//-Harrison, C. L.-//-Moran, L. J.-//-Strauss, B. J.-//-Teede, H. J. | Effects of exercise on insulin resistance and body composition in overweight and obese women with and without polycystic ovary syndrome | Different study design |  |
| 2009 | Brown, A. J.-//-Setji, T. L.-//-Sanders, L. L.-//-Lowry, K. P.-//-Otvos, J. D.-//-Kraus, W. E.-//-Svetkey, P. L. | Effects of exercise on lipoprotein particles in women with polycystic ovary syndrome | Different study design |  |
| 2022 | Rao, M.-//-Khan, A. A.-//-Adnan, Q. U. A. | Effects of high-intensity interval training and strength training on levels of testosterone and physical activity among women with polycystic ovary syndrome | Different study design |  |
| 2019 | Kogure, G. S.-//-Miranda-Furtado, C. L.-//-Pedroso, D. C. C.-//-Ribeiro, V. B.-//-Eiras, M. C.-//-Silva, R. C.-//-Caetano, L. C.-//-Ferriani, R. A.-//-Calado, R. T.-//-dos Reis, R. M. | Effects of Progressive Resistance Training on Obesity Indices in Polycystic Ovary Syndrome and the Relationship With Telomere Length | Different study design |  |
| 2017 | Al-Eisa, E.-//-Gabr, S. A.-//-Alghadir, A. H. | Effects of supervised aerobic training on the levels of anti-Mullerian hormone and adiposity measures in women with normo-ovulatory and polycystic ovary syndrome | Different study design |  |
| 2016 | Nasrekani, Z. A.-//-Fathi, M. | Efficacy of 12 weeks aerobic training on body composition, aerobic power and some women-hormones in polycystic ovary syndrome infertile women | Different study design |  |
| 2022 | Niranjani, S.-//-Bhuvaneswari, G.-//-Hemamalini, M.-//-Vijayalakshmi, R. | Efficacy of cinnamon, exercise and counselling (Multi – interventional Package) on insulin resistance among young girls with Polycystic Ovarian Syndrome | Different study design |  |
| 2020 | Zheng, S.-//-Zhang, Y.-//-Li, R.-//-Xue, J.-//-Shen, H. | Efficacy of internet-based intensive weight management or exenatide treatment on weight loss and metabolism in overweight/obese pcos | Different study design |  |
| 2022 | Kaur, I.-//-Suri, V.-//-Sachdeva, N.-//-Rana, S. V.-//-Medhi, B.-//-Sahni, N.-//-Ahire, J.-//-Singh, A. | Efficacy of multi-strain probiotic along with dietary and lifestyle modifications on polycystic ovary syndrome: a randomised, double-blind placebo-controlled study | Different study design |  |
| 2016 | Hulchiy, M.-//-Nybacka, Å-//-Sahlin, L.-//-Hirschberg, A. L. | Endometrial expression of estrogen receptors and the androgen receptor in women with polycystic ovary syndrome: A lifestyle intervention study | Different study design |  |
| 2019 | Mishra, A.-//-Sharma, R.-//-Mittal, P.-//-Kapoor, R.-//-Srivastav, S. | Evaluating exercise challenge to validate cardiac autonomic dysfunction in lean PCOS phenotype | Different study design |  |
| 2019 | Samadi, Z.-//-Bambaeichi, E.-//-Valiani, M.-//-Shahshahan, Z. | Evaluation of Changes in Levels of Hyperandrogenism, Hirsutism and Menstrual Regulation After a Period of Aquatic High Intensity Interval Training in Women with Polycystic Ovary Syndrome | Different study design |  |
| 2012 | Eleftheriadou, M.-//-Michala, L.-//-Stefanidis, K.-//-Iliadis, I.-//-Lykeridou, A.-//-Antsaklis, A. | Exercise and sedentary habits among adolescents with PCOS | Different study design |  |
| 2008 | Giallauria, F.-//-Palomba, S.-//-Maresca, L.-//-Vuolo, L.-//-Tafuri, D.-//-Lombardi, G.-//-Colao, A.-//-Vigorito, C.-//-Francesco, O. | Exercise training improves autonomic function and inflammatory pattern in women with polycystic ovary syndrome (PCOS) | Different study design |  |
| 2013 | Sprung, V. S.-//-Cuthbertson, D. J.-//-Pugh, C. J. A.-//-Aziz, N.-//-Kemp, G. J.-//-Daousi, C.-//-Green, D. J.-//-Cable, N. T.-//-Jones, H. | Exercise Training in Polycystic Ovarian Syndrome Enhances Flow-Mediated Dilation in the Absence of Changes in Fatness | Different study design |  |
| 2019 | Dantas, W. S.-//-Neves, W. D.-//-Gil, S.-//-Barcellos, C. R. G.-//-Rocha, M. P.-//-de Sá-Pinto, A. L.-//-Roschel, H.-//-Gualano, B. | Exercise-induced anti-inflammatory effects in overweight/obese women with polycystic ovary syndrome | Different study design |  |
| 2017 | Scott, D.-//-Harrison, C. L.-//-Hutchison, S.-//-De Courten, B.-//-Stepto, N. K. | Exploring factors related to changes in body composition, insulin sensitivity and aerobic capacity in response to a 12-week exercise intervention in overweight and obese women with and without polycystic ovary syndrome | Different study design |  |
| 2015 | Dantas, W. S.-//-Marcondes, J. A.-//-Shinjo, S. K.-//-Perandini, L. A.-//-Zambelli, V. O.-//-Neves, W. D.-//-Barcellos, C. R.-//-Rocha, M. P.-//-Yance Vdos, R.-//-Pereira, R. T.-//-Murai, I. H.-//-Pinto, A. L.-//-Roschel, H.-//-Gualano, B. | GLUT4 translocation is not impaired after acute exercise in skeletal muscle of women with obesity and polycystic ovary syndrome | Different study design |  |
| 2019 | King, A. K.-//-McGill-Meeks, K.-//-Beller, J. P.-//-Solorzano, C. M. B. | Go Girls!-Dance-Based Fitness to Increase Enjoyment of Exercise in Girls at Risk for PCOS | Different study design |  |
| 2010 | Stachenfeld, N. S.-//-Yeckel, C. W.-//-Taylor, H. S. | Greater exercise sweating in obese women with polycystic ovary syndrome compared to obese controls | Different study design |  |
| 2011 | Pasquali, R.-//-Gambineri, A.-//-Cavazza, C.-//-Ibarra Gasparini, D.-//-Ciampaglia, W.-//-Cognigni, G. E.-//-Pagotto, U. | Heterogeneity in the responsiveness to long-term lifestyle intervention and predictability in obese women with polycystic ovary syndrome | Different study design |  |
| 2015 | Almenning, I.-//-Rieber-Mohn, A.-//-Garnaes, K. K.-//-Lundgren, K. M.-//-Lovik, T. S.-//-Trine Moholdt, T. | High intensity interval training versus strength training to improve cardiovascular risk factors in women with polycystic ovary syndrome. A randomized controlled trial | Different study design |  |
| 2016 | Covington, J. D.-//-Tam, C. S.-//-Pasarica, M.-//-Redman, L. M. | Higher circulating leukocytes in women with PCOS is reversed by aerobic exercise | Different study design |  |
| 2022 | Kiel, I. A.-//-Lionett, S.-//-Parr, E. B.-//-Jones, H.-//-Røset, M. A. H.-//-Salvesen, Ø-//-Hawley, J. A.-//-Vanky, E.-//-Moholdt, T. | High-Intensity Interval Training in Polycystic Ovary Syndrome: A Two-Center, Three-Armed Randomized Controlled Trial | Different study design |  |
| 2022 | Patten, R. K.-//-McIlvenna, L. C.-//-Levinger, I.-//-Garnham, A. P.-//-Shorakae, S.-//-Parker, A. G.-//-McAinch, A. J.-//-Rodgers, R. J.-//-Hiam, D.-//-Moreno-Asso, A.-//-Stepto, N. K. | High-intensity training elicits greater improvements in cardio-metabolic and reproductive outcomes than moderate-intensity training in women with polycystic ovary syndrome: a randomized clinical trial | Different study design |  |
| 1993 | Jaatinen, T. A.-//-Anttila, L.-//-Erkkola, R.-//-Koskinen, P.-//-Laippala, P.-//-Ruutiainen, K.-//-Scheinin, M.-//-Irjala, K. | Hormonal responses to physical exercise in patients with polycystic ovarian syndrome | Different study design |  |
| 2018 | Kogure, G. S.-//-Silva, R. C.-//-Miranda-Furtado, C. L.-//-Ribeiro, V. B.-//-Pedroso, D. C. C.-//-Melo, A. S.-//-Ferriani, R. A.-//-Reis, R. M. D. | Hyperandrogenism Enhances Muscle Strength After Progressive Resistance Training, Independent of Body Composition, in Women With Polycystic Ovary Syndrome | Different study design |  |
| 2014 | Asante, A.-//-Konopka, A. R.-//-Stewart, E. A.-//-Coddington, C. C.-//-Nair, S. K. | Impact of Aerobic Exercise on Mitochondrial Function and Insulin Sensitivity in Women with Polycystic Ovary Syndrome - A Randomized Controlled Trial | Different study design |  |
| 2011 | Jedel, E.-//-Labrie, F.-//-Odén, A.-//-Holm, G.-//-Nilsson, L.-//-Janson, P. O.-//-Lind, A. K.-//-Ohlsson, C.-//-Stener-Victorin, E. | Impact of electro-acupuncture and physical exercise on hyperandrogenism and oligo/amenorrhea in women with polycystic ovary syndrome: a randomized controlled trial | Different study design |  |
| 2020 | Selvaraj, V.-//-Vanitha, J.-//-Dhanaraj, F. M.-//-Sekar, P.-//-Babu, A. R. | Impact of yoga and exercises on polycystic ovarian syndrome risk among adolescent schoolgirls in South India | Different study design |  |
| 2020 | Vince, R. V.-//-Kirk, R. J.-//-Aye, M. M.-//-Atkin, S. L.-//-Madden, L. A. | Impaired heat shock protein 72 expression in women with polycystic ovary syndrome following a supervised exercise programme | Different study design |  |
| 2011 | Vind, B. F.-//-Pehmoller, C.-//-Treebak, J. T.-//-Birk, J. B.-//-Hey-Mogensen, M.-//-Beck-Nielsen, H.-//-Zierath, J. R.-//-Wojtaszewski, J. F. P.-//-Hojlund, K. | Impaired insulin-induced site-specific phosphorylation of TBC1 domain family, member 4 (TBC1D4) in skeletal muscle of type 2 diabetes patients is restored by endurance exercise-training | Different study design |  |
| 2021 | Wu, X.-//-Wu, H.-//-Sun, W.-//-Wang, C. | Improvement of anti-Müllerian hormone and oxidative stress through regular exercise in Chinese women with polycystic ovary syndrome | Different study design |  |
| 2021 | de Loos, Alpd-//-Jiskoot, G.-//-Timman, R.-//-Beerthuizen, A.-//-Busschbach, J. J. V.-//-Laven, J. S. E. | Improvements in PCOS characteristics and phenotype severity during a randomized controlled lifestyle intervention | Different study design |  |
| 2017 | Nybacka, A.-//-Hellstrom, P. M.-//-Hirschberg, A. L. | Increased fibre and reduced trans fatty acid intake are primary predictors of metabolic improvement in overweight polycystic ovary syndromeSubstudy of randomized trial between diet, exercise and diet plus exercise for weight control | Different study design |  |
| 2017 | Nybacka, Å-//-Hellström, P. M.-//-Hirschberg, A. L. | Increased fibre and reduced trans fatty acid intake are primary predictors of metabolic improvement in overweight polycystic ovary syndrome-Substudy of randomized trial between diet, exercise and diet plus exercise for weight control | Different study design |  |
| 2020 | Zhang, B.-//-Zhou, W.-//-Shi, Y.-//-Zhang, J.-//-Cui, L.-//-Chen, Z. J. | Lifestyle and environmental contributions to ovulatory dysfunction in women of polycystic ovary syndrome | Different study design |  |
| 2021 | Liu, C.-//-Zhang, L.-//-Zheng, W.-//-Liang, X.-//-Zhang, L.-//-Tian, Z.-//-Li, G. | Lifestyle Intervention for Overweight/Obese Pregnant Women with Polycystic Ovarian Syndrome: Lessons and Challenges | Different study design |  |
| 2014 | Ujvari, D.-//-Hulchiy, M.-//-Calaby, A.-//-Nybacka, A.-//-Bystrom, B.-//-Hirschberg, A. L. | Lifestyle intervention up-regulates gene and protein levels of molecules involved in insulin signaling in the endometrium of overweight/obese women with polycystic ovary syndrome | Different study design |  |
| 2023 | Patten, R. K.-//-Bourke, M.-//-McIlvenna, L. C.-//-Moreno-Asso, A.-//-Woessner, M. N.-//-Stepto, N. K.-//-Parker, A. | Longitudinal affective response to high-intensity interval training and moderate-intensity continuous training in overweight women with polycystic ovary syndrome: A randomised trial | Different study design |  |
| 2015 | McBreairty, L.-//-Zello, G.-//-Rooke, J.-//-Serrao, S.-//-Pierson, R.-//-Chizen, D.-//-Chilibeck, P. | Long-term effect of a pulse-based diet and exercise training intervention on body composition and dietary intake in women with polycystic ovarian syndrome | Different study design |  |
| 2009 | Stener-Victorin, E.-//-Jedel, E.-//-Janson, P. O.-//-Sverrisdottir, Y. B. | Low-frequency electroacupuncture and physical exercise decrease high muscle sympathetic nerve activity in polycystic ovary syndrome | Different study design |  |
| 2020 | Hansen, S. L.-//-Bojsen-Møller, K. N.-//-Lundsgaard, A. M.-//-Hendrich, F. L.-//-Nilas, L.-//-Sjøberg, K. A.-//-Hingst, J. R.-//-Serup, A. K.-//-Olguín, C. H.-//-Carl, C. S.-//-Wernblad, L. F.-//-Henneberg, M.-//-Lustrup, K. M.-//-Hansen, C.-//-Jensen, T. E.-//-Madsbad, S.-//-Wojtaszewski, J. F. P.-//-Richter, E. A.-//-Kiens, B. | Mechanisms underlying absent training-induced improvement in insulin action in lean, hyperandrogenic women with polycystic ovary syndrome | Different study design |  |
| 2008 | Orio, F.-//-Giallauria, F.-//-Palomba, S.-//-Manguso, F.-//-Orio, M.-//-Tafuri, D.-//-Lombardi, G.-//-Carmina, E.-//-Colao, A.-//-Vigorito, C. | Metabolic and cardiopulmonary effects of detraining after a structured exercise training programme in young PCOS women | Different study design |  |
| 2022 | de Loos, A. D.-//-Jiskoot, G.-//-Beerthuizen, A.-//-Busschbach, J.-//-Laven, J. | Metabolic health during a randomized controlled lifestyle intervention in women with PCOS | Different study design |  |
| 2021 | Dietz De Loos, A.-//-Jiskoot, G.-//-Beerthuizen, A.-//-Van Busschbach, J.-//-Laven, J. | Metabolic syndrome prevalence and severity during a randomized controlled three-component lifestyle intervention in women with PCOS | Different study design |  |
| 2019 | Halama, A.-//-Aye, M. M.-//-Dargham, S. R.-//-Kulinski, M.-//-Suhre, K.-//-Atkin, S. L. | Metabolomics of Dynamic Changes in Insulin Resistance Before and After Exercise in PCOS | Different study design |  |
| 2017 | Sant'Anna, E. M.-//-Paiva, S. P. C.-//-Santos, R. P.-//-Rodrigues, A. M.-//-Nery, S. F.-//-Maia, F. P.-//-Ferreira, A. V. M.-//-Reis, F. | Mindfulness-based intervention for lifestyle modification and weight loss in infertile women: Randomized controlled trial | Different study design |  |
| 2013 | Sprung, V. S.-//-Cuthbertson, D. J.-//-Pugh, C. J.-//-Daousi, C.-//-Atkinson, G.-//-Aziz, N. F.-//-Kemp, G. J.-//-Green, D. J.-//-Cable, N. T.-//-Jones, H. | Nitric oxide-mediated cutaneous microvascular function is impaired in polycystic ovary sydrome but can be improved by exercise training | Different study design |  |
| 2022 | Jackson, B.-//-Kishan, R.-//-Mullins, C.-//-Mathew, M.-//-Kim, S.-//-Huang, J. C.-//-Phy, J. L. | NUTRITIONAL EDUCATION (FACE-TO-FACE AND VIDEO INSTRUCTION) FOR POLYCYSTIC OVARY SYNDROME RESULTS IN GREATER REDUCTION IN BMI AND HEMOGLOBIN A1C THAN CALORIC RESTRICTION, EXERCISE AND METFORMIN | Different study design |  |
| 2016 | Orio, F.-//-Muscogiuri, G.-//-Giallauria, F.-//-Savastano, S.-//-Bottiglieri, P.-//-Tafuri, D.-//-Predotti, P.-//-Colarieti, G.-//-Colao, A.-//-Palomba, S. | Oral contraceptives versus physical exercise on cardiovascular and metabolic risk factors in women with polycystic ovary syndrome: a randomized controlled trial | Different study design |  |
| 2022 | Mohsin, A. N.-//-Al-Selmi, Ladh | Physical-Nutritional program effect in women with Polycystic Ovary Syndrome (PCOS) | Different study design |  |
| 2012 | Joham, A. E.-//-Teede, H. J.-//-Hutchison, S. K.-//-Stepto, N. K.-//-Harrison, C. L.-//-Strauss, B. J.-//-Paul, E.-//-Watt, M. J. | Pigment epithelium-derived factor, insulin sensitivity, and adiposity in polycystic ovary syndrome: impact of exercise training | Different study design |  |
| 2012 | Nyback, A.-//-Fabri, F.-//-Hellstrom, P. M.-//-Hirschberg, A. L.-//-Stahle, A. | Plasma Anti-Mullerian Hormone (AMH) levels correlate and predict the response to lifestyle intervention in polycystic ovarian syndrome | Different study design |  |
| 2015 | Nybacka, A.-//-Hellstrom, P. M.-//-Hirschberg, A. L. | Plasma vitamin D in response to dietary management and/or physical exercise in obese women with polycystic ovary syndrome | Different study design |  |
| 2016 | Ribeiro, V. B.-//-Kogure, G. S.-//-Reis, R. M.-//-Gastaldi, A. C.-//-D. E. Araújo JE-//-Mazon, J. H.-//-Borghi, A.-//-Souza, H. C. | Polycystic Ovary Syndrome Presents Higher Sympathetic Cardiac Autonomic Modulation that is not altered by Strength Training | Different study design |  |
| 2021 | Jiskoot, G.-//-Dietz De Loos, A.-//-Timman, R.-//-Beerthuizen, A.-//-Busschbach, J.-//-Laven, J. | Prediction of weight loss and drop-out in a lifestyle intervention in women with pcos: a randomized controlled trial | Different study design |  |
| 2023 | Dietz de Loos, A.-//-Jiskoot, G.-//-Louwers, Y.-//-Beerthuizen, A.-//-Busschbach, J.-//-Laven, J. | Pregnancy Outcomes in Women with PCOS: Follow-Up Study of a Randomized Controlled Three-Component Lifestyle Intervention | Different study design |  |
| 2019 | Huang, D.-//-Greenwood, E. A.-//-Kao, C. N.-//-Quinn, M.-//-Cedars, M. I.-//-Huddleston, H. G. | Prevalence and predictors of adequate physical activity in a multiethnic polycystic ovary syndrome patient population | Different study design |  |
| 2021 | Benharrat, L. I.-//-Senouci, A.-//-Benhabib, W.-//-Mekki, K. | Prevalence of metabolic syndrome in women with polycystic ovary syndrome: Relationship with lifestyle and cardiometabolic biomarkers | Different study design |  |
| 2017 | Paulson, M.-//-Sahlin, L.-//-Hirschberg, A. L. | Progesterone receptors and proliferation of the endometrium in obese women with polycystic ovary syndrome-a lifestyle intervention study | Different study design |  |
| 2019 | Tiwari, N.-//-Pasrija, S.-//-Jain, S. | Randomised controlled trial to study the efficacy of exercise with and without metformin on women with polycystic ovary syndrome | Different study design |  |
| 2011 | Nybacka, Å-//-Carlström, K.-//-Ståhle, A.-//-Nyrén, S.-//-Hellström, P. M.-//-Hirschberg, A. L. | Randomized comparison of the influence of dietary management and/or physical exercise on ovarian function and metabolic parameters in overweight women with polycystic ovary syndrome | Different study design |  |
| 2021 | Jerobin, J.-//-Ramanjaneya, M.-//-Bettahi, I.-//-Parammal, R.-//-Siveen, K. S.-//-Alkasem, M.-//-Aye, M.-//-Sathyapalan, T.-//-Skarulis, M.-//-Atkin, S. L.-//-Abou-Samra, A. B. | Regulation of circulating CTRP-2/CTRP-9 and GDF-8/GDF-15 by intralipids and insulin in healthy control and polycystic ovary syndrome women following chronic exercise training | Different study design |  |
| 2016 | Kogure, G. S.-//-Miranda-Furtado, C. L.-//-Silva, R. C.-//-Melo, A. S.-//-Ferriani, R. A.-//-De Sá, M. F.-//-Dos Reis, R. M. | Resistance Exercise Impacts Lean Muscle Mass in Women with Polycystic Ovary Syndrome | Different study design |  |
| 2008 | Cosar, E.-//-Köken, G.-//-Sahin, F. K.-//-Akgün, L.-//-Uçok, K.-//-Genç, A.-//-Yilmazer, M. | Resting metabolic rate and exercise capacity in women with polycystic ovary syndrome | Different study design |  |
| 1999 | Huber-Buchholz, M. M.-//-Carey, D. G. P.-//-Norman, R. J. | Restoration of reproductive potential by lifestyle modification in obese polycystic ovary syndrome: Role of insulin sensitivity and luteinizing hormone | Different study design |  |
| 2016 | McBreairty, L. E.-//-Zello, G. A.-//-Gordon, J. J.-//-Chizen, D. R.-//-Chilibeck, P. D. | Sarcopenic obesity in women with polycystic ovary syndrome: effect of a pulse based-diet and exercise intervention | Different study design |  |
| 2013 | Saremi, A.-//-Shavandi, N.-//-Karamali, M.-//-Kazemi, M. | Serum level of anti-mullerian hormone after exercise training in women with polycystic ovary syndrome: A randomized controlled trial | Different study design |  |
| 2021 | Ribeiro, V. B.-//-Pedroso, D. C. C.-//-Kogure, G. S.-//-Lopes, I. P.-//-Santana, B. A.-//-Dutra de Souza, H. C.-//-Ferriani, R. A.-//-Calado, R. T.-//-Furtado, C. L. M.-//-Reis, R. M. D. | Short-Term Aerobic Exercise Did Not Change Telomere Length While It Reduced Testosterone Levels and Obesity Indexes in PCOS: A Randomized Controlled Clinical Trial Study | Different study design |  |
| 2022 | Bennett, C. J.-//-Mansfield, D. R.-//-Mo, L.-//-Joham, A. E.-//-Cain, S. W.-//-Blumfield, M. L.-//-Hodge, A. M.-//-Moran, L. J. | Sleep disturbances may influence lifestyle behaviours in women with self-reported polycystic ovary syndrome | Different study design |  |
| 2022 | Burnik Papler, T.-//-Abdulkhalikova, D.-//-Jancar, N.-//-Sustarsic, A.-//-Vrtacnik Bokal, E. | Spontaneous pregnancy rates after the weight loss program in infertile PCOS women with obesity | Different study design |  |
| 2010 | Palomba, S.-//-Giallauria, F.-//-Falbo, A.-//-Russo, T.-//-Grieco, A.-//-Colao, A.-//-Lombardi, G.-//-Orio, F. | Structured exercise training plus hypocaloric diet improves ovarian sensitivity to clomiphene citrate in polycystic ovary syndrome (PCOS) patients | Different study design |  |
| 2022 | Woodward, A.-//-Broom, D.-//-Dalton, C.-//-Metwally, M.-//-Klonizakis, M. | Supervised Aerobic Exercise Training and Increased Lifestyle Physical Activity to Reduce Cardiovascular Disease Risk for Women With Polycystic Ovary Syndrome: A Randomized Controlled Feasibility Trial | Different study design |  |
| 2013 | Miranda-Furtado, C. L.-//-Ramos, F. K. P.-//-Kogure, G. S.-//-Silva, R. C.-//-Calado, R. T.-//-Reis, R. M. | Telomere lenght in polycystic ovary syndrome: Does it change after physical exercise resistance? | Different study design |  |
| 2017 | Hmedeh, C.-//-Ghazeeri, G.-//-Tinworth, L.-//-Tewfik, I. | The effect of 6 months weight -loss/maintenance on anthropometric, biochemical and psychological profile in Lebanese PCOS women: A prospective randomised control study | Different study design |  |
| 2019 | Javid, N. M.-//-Behpour, N.-//-Tadibi, V. | The Effect of a 16-week Home-based Aerobic Exercise Program on Serum High-sensitivity C-Reactive Protein (Hs-CRP) and Insulin Resistance in Polycystic Ovary Syndrome | Different study design |  |
| 2008 | Thomson, R. L.-//-Buckley, J. D.-//-Noakes, M.-//-Clifton, P. M.-//-Norman, R. J.-//-Brinkworth, G. D. | The effect of a hypocaloric diet with and without exercise training on body composition, cardiometabolic risk profile, and reproductive function in overweight and obese women with polycystic ovary syndrome | Different study design |  |
| 2015 | Sweatt, K.-//-Ovalle, F.-//-Azziz, R.-//-Gower, B. | The effect of diet and exercise in women with polycystic ovary syndrome | Different study design |  |
| 2021 | Shahidi, F.-//-Gaeini, A. A.-//-Afghan, M.-//-Hosseini, R. | The Effect of Eight Weeks of High-intensity Interval Training on Serum Levels of VCAM-1 and E-selectin in Women with Polycystic Ovary Syndrome | Different study design |  |
| 2022 | Afghan, Marjan-//-Shahidi, Fereshteh-//-Gaeini, Abbasali-//-Hosseini, Roya | The Effect of Eight Weeks of Moderate-Intensity Continious Training on P- and QT Dispersion in ECG of Women with Polycystic Ovary Syndrome | Different study design |  |
| 2022 | Nasiri, M.-//-Monazzami, A.-//-Alavimilani, S.-//-Asemi, Z. | The Effect of High Intensity Intermittent and Combined (Resistant and Endurance) Trainings on Some Anthropometric Indices and Aerobic Performance in Women with Polycystic Ovary Syndrome: A Randomized Controlled Clinical Trial Study | Different study design |  |
| 2016 | Nagelberg, J.-//-Burks, H.-//-Mucowski, S.-//-Shoupe, D. | The effect of home exercise on ovulation induction using clomiphene citrate in overweight underserved women with polycystic ovarian syndrome | Different study design |  |
| 2019 | Bahrami, H.-//-Mohseni, M.-//-Amini, L.-//-Karimian, Z. | The effect of six weeks yoga exercises on quality of life in infertile women with polycystic ovary syndrome (Pcos) | Different study design |  |
| 2022 | Aktaş, HŞ-//-Uzun, Y. E.-//-Kutlu, O.-//-Pençe, H. H.-//-Özçelik, F.-//-Çil, EÖ-//-Irak, L.-//-Altun, Ö-//-Özcan, M.-//-Özsoy, N.-//-Aydın Yoldemir, Ş-//-Kalyon, S.-//-Arman, Y.-//-Tükek, T. | The effects of high intensity-interval training on vaspin, adiponectin and leptin levels in women with polycystic ovary syndrome | Different study design |  |
| 2022 | Gorczyca, A. M.-//-Steger, F. L.-//-Ptomey, L. T.-//-Montgomery, R. N.-//-Mickelsen, R.-//-Smith, P.-//-Donnelly, J. E.-//-Marsh, C. A. | The impact of a group based, remotely delivered weight loss intervention in women with polycystic ovary syndrome on ovulation, quality of life and body composition | Different study design |  |
| 2012 | Harrison, C. L.-//-Stepto, N. K.-//-Hutchison, S. K.-//-Teede, H. J. | The impact of intensified exercise training on insulin resistance and fitness in overweight and obese women with and without polycystic ovary syndrome | Different study design |  |
| 2021 | Mohseni, M.-//-Eghbali, M.-//-Bahrami, H.-//-Dastaran, F.-//-Amini, L. | Yoga Effects on Anthropometric Indices and Polycystic Ovary Syndrome Symptoms in Women Undergoing Infertility Treatment: A Randomized Controlled Clinical Trial | Different study design |  |
| 2021 | Yuvarani, G.-//-Banu, I. S.-//-Tharani, G.-//-Kamatchi, K.-//-Princy, F. | Comparison of Short-Term Structured Exercise in Non-Over Weight and Overweight Subjects with Polycystic Ovarian Syndrome | Insufficient data reported |  |
| 2013 | Nidhi, R.-//-Padmalatha, V.-//-Nagarathna, R.-//-Amritanshu, R. | Effect of Yoga Program on Quality of Life in Adolescent Polycystic Ovarian Syndrome: A Randomized Control Trial | Insufficient data reported |  |
| 2018 | Patil, V. R.-//-Thangavelu, P. D.-//-Jagtap, V. K. | Effectiveness of lifestyle modification on weightloss and quality of life in obese women with polycystic ovarian syndrome | Insufficient data reported |  |
| 2016 | Ramos, F. K.-//-Lara, L. A.-//-Kogure, G. S.-//-Silva, R. C.-//-Ferriani, R. A.-//-Silva de Sá, M. F.-//-Reis, R. M. | Quality of Life in Women with Polycystic Ovary Syndrome after a Program of Resistance Exercise Training | Insufficient data reported |  |
| 2018 | Barbosa, C. I. B.-//-Caldas, C. E.-//-Fernando, F. Jl-//-Browne, R. A. V.-//-Ferezini De Sa, J. C.-//-Keith, S. N. | Aerobic Training Improves Quality of Life in Women with Polycystic Ovary Syndrome | Different study design |  |
| 2018 | Amini, L.-//-Mohseni, M.-//-Bahrami, H.-//-Haghani, H. | Can yoga exercises improve in vitro fertilization outcomes in women with polycystic ovary syndrome? | Unverified citation | unlocated due to no author |
| 2017 | Akbari, S. A. A. | Comparison of lifestyle between women with and without polycystic ovary syndrome | Different study design |  |
| 2013 | Mirfeizi, M. | Comparison of the effects of a diet and physical activity trial in obese women with polycystic ovary syndrome | Different study design |  |
| 2014 | Distelmaier, K.-//-Asante, A.-//-Konopka, A.-//-Shankarappa, M.-//-Amols, M.-//-Nair, K. | Effect of aerobic exercise on HDL function in women with polycystic ovary syndrome: a randomised controlled trial | Different study design |  |
| 2019 | Hanafy, H. M.-//-Awad, M. A.-//-Kamel, H. E. H.-//-Mohamed, D. S. | Effect of aerobic exercise on non-obese adolescent girls with polycystic ovarian syndrome | Different population |  |
| 2005 | Brown, A. J.-//-Aiken, L. B.-//-Setji, T.-//-Sanders, L.-//-Kraus, W. E.-//-Svetkey, L. P. | Effect of exercise without weight loss on insulin resistance in women with polycystic ovary syndrome: a randomized controlled study | Different study design |  |
| 2010 | Jedel, E.-//-Holm, G.-//-Labrie, F.-//-Oden, A.-//-Nilsson, L.-//-Janson, P. O.-//-Ohlsson, C.-//-Stener-Victorin, E. | Effect of Low-Frequency Electro-Acupuncture on Serum Testosterone and Menstrual Pattern in Women with Polycystic Ovary Syndrome Compared to Physical Exercise: Randomised Controlled Trial | Unverified citation | unlocated due to no author |
| 2017 | Manjrekar, G.-//-Bhide, G. | Effect of physical activity intervention on overall physical ftness in women with and without Polycystic Ovarian Syndrome (PCOS) | Different study design |  |
| 2022 | Sonntag, B. | Influence of exercise intensity on markers of polycystic ovarian syndrome - a randomized controlled study | Unverified citation | unlocated due to no author |
| 2012 | Arabipoor, A.-//-Kiani, K. | The role of lifestyle modification in management of overweight infertile women with polycystic ovary syndrome | Abstract/Protocol/Opinion/Review |  |
| 2015 | Diane, A.-//-Kupreeva, M.-//-Borthwick, F.-//-Proctor, S. D.-//-Pierce, W. D.-//-Vine, D. F. | Cardiometabolic and reproductive benefits of early dietary energy restriction and voluntary exercise in an obese PCOS-prone rodent model | Preclinical study |  |
| 2001 | Graham-Thiers, P. M.-//-Kronfeld, D. S.-//-Kline, K. A.-//-Sklan, D. J. | Dietary protein restriction and fat supplementation diminish the acidogenic effect of exercise during repeated sprints in horses | Preclinical study |  |
| 2022 | Zhang, Y.-//-Chen, D.-//-Wang, D.-//-Wang, L.-//-Weng, Y.-//-Wang, H.-//-Wu, X.-//-Wang, Y. | Moderate Aerobic Exercise Regulates Follicular Dysfunction by Initiating Brain-Derived Neurotrophic Factor (BDNF)-Mediated Anti-Apoptotic Signaling Pathways in Polycystic Ovary Syndrome | Preclinical study |  |
| 2014 | Wu, C.-//-Lin, F.-//-Qiu, S.-//-Jiang, Z. | The characterization of obese polycystic ovary syndrome rat model suitable for exercise intervention | Preclinical study |  |
| 2020 | Kazemi, M.-//-McBreairty, L. E.-//-Zello, G. A.-//-Pierson, R. A.-//-Gordon, J. J.-//-Serrao, S. B.-//-Chilibeck, P. D.-//-Chizen, D. R. | A pulse-based diet and the Therapeutic Lifestyle Changes diet in combination with health counseling and exercise improve health-related quality of life in women with polycystic ovary syndrome: secondary analysis of a randomized controlled trial | Secondary report |  |
| 2018 | Lopes, I. P.-//-Ribeiro, V. B.-//-Reis, R. M.-//-Silva, R. C.-//-Dutra de Souza, H. C.-//-Kogure, G. S.-//-Ferriani, R. A.-//-Silva Lara, L. A. D. | Comparison of the Effect of Intermittent and Continuous Aerobic Physical Training on Sexual Function of Women With Polycystic Ovary Syndrome: Randomized Controlled Trial | Secondary report |  |
| 2019 | Jiskoot, L.-//-Timman, R.-//-Beerthuizen, A.-//-Dietz De Loos, A.-//-Busschbach, J.-//-Laven, J. | Effects of a three-component lifestyle intervention on emotional well-being in women with PCOS | Secondary report |  |
| 2012 | Stener-Victorin, E.-//-Baghaei, F.-//-Holm, G.-//-Janson, P. O.-//-Olivecrona, G.-//-Lönn, M.-//-Mannerås-Holm, L. | Effects of acupuncture and exercise on insulin sensitivity, adipose tissue characteristics, and markers of coagulation and fibrinolysis in women with polycystic ovary syndrome: secondary analyses of a randomized controlled trial | Secondary report |  |
| 2020 | Stepto, N. K.-//-Hiam, D.-//-Gibson-Helm, M.-//-Cassar, S.-//-Harrison, C. L.-//-Hutchison, S. K.-//-Joham, A. E.-//-Canny, B. J.-//-Moreno-Asso, A.-//-Strauss, B. J.-//-Hatzirodos, N.-//-Rodgers, R. J.-//-Teede, H. J. | Exercise and insulin resistance in PCOS: Muscle insulin signalling and fibrosis | Secondary report |  |
| 2020 | Jiskoot, G.-//-Dietz de Loos, A.-//-Beerthuizen, A.-//-Timman, R.-//-Busschbach, J.-//-Laven, J. | Long-term effects of a three-component lifestyle intervention on emotional well-being in women with Polycystic Ovary Syndrome (PCOS): A secondary analysis of a randomized controlled trial | Secondary report |  |
| 2021 | Nl | Long-term follow up of overweight and obese women with PCOS who participated in a randomized controlled three-component lifestyle study | Secondary report |  |
| 2019 | Moran, L. J.-//-Noakes, M.-//-Clifton, P.-//-Buckley, J.-//-Brinkworth, G.-//-Thomson, R.-//-Norman, R. J. | Predictors of Lifestyle Intervention Attrition or Weight Loss Success in Women with Polycystic Ovary Syndrome Who Are Overweight or Obese | Secondary report |  |
| 2015 | Leonhardt, H.-//-Hellström, M.-//-Gull, B.-//-Lind, A. K.-//-Nilsson, L.-//-Janson, P. O.-//-Stener-Victorin, E. | Serum anti-Müllerian hormone and ovarian morphology assessed by magnetic resonance imaging in response to acupuncture and exercise in women with polycystic ovary syndrome: secondary analyses of a randomized controlled trial | Secondary report |  |
| 2013 | Nybacka, Å-//-Carlström, K.-//-Fabri, F.-//-Hellström, P. M.-//-Hirschberg, A. L. | Serum antimüllerian hormone in response to dietary management and/or physical exercise in overweight/obese women with polycystic ovary syndrome: secondary analysis of a randomized controlled trial | Secondary report |  |
| 2012 | Thomson, R. L.-//-Brinkworth, G. D.-//-Noakes, M.-//-Clifton, P. M.-//-Norman, R. J.-//-Buckley, J. D. | The effect of diet and exercise on markers of endothelial function in overweight and obese women with polycystic ovary syndrome | Secondary report |  |
| 2011 | Thomson, R. L.-//-Brinkworth, G. D.-//-Noakes, M.-//-Clifton, P. M.-//-Norman, R. J.-//-Buckley, J. D. | The effect of diet and exercise on vascular function in overweight and obese women with polycystic ovary syndrome | Secondary report |  |
| 2018 | Costa, E. C.-//-De Sa, J. C. F.-//-Stepto, N. K.-//-Costa, I. B. B.-//-Farias, L. F.-//-Moreira, S. D. T.-//-Soares, E. M. M.-//-Lemos, Tmam-//-Browne, R. A. V.-//-Azevedo, G. D. | Aerobic Training Improves Quality of Life in Women with Polycystic Ovary Syndrome | Duplicate |  |
| 2022 | Jiskoot, G.-//-de Loos, A. D.-//-Timman, R.-//-Beerthuizen, A.-//-Laven, J.-//-Busschbach, J. | Changes in eating behavior through lifestyle treatment in women with polycystic ovary syndrome (PCOS): a randomized controlled trial | Different study design |  |
| 2021 | Ribeiro, V. B.-//-Lopes, I. P.-//-Dos Reis, R. M.-//-Silva, R. C.-//-Mendes, M. C.-//-Melo, A. S.-//-de Souza, H. C. D.-//-Ferriani, R. A.-//-Kogure, G. S.-//-Lara, Lads | Continuous versus intermittent aerobic exercise in the improvement of quality of life for women with polycystic ovary syndrome: A randomized controlled trial | Different study design | Different outcomes, quality of life |
| 2012 | Nidhi, R.-//-Padmalatha, V.-//-Nagarathna, R.-//-Amritanshu, R. | Effect of holistic yoga program on anxiety symptoms in adolescent girls with polycystic ovarian syndrome: A randomized control trial | Duplicate |  |
| 2022 | Niranjani, S.-//-Bhuvaneswari, G.-//-Hemamalini, M.-//-Vijayaraghavan, R. | Effectiveness of cinnamon, exercise and counselling on hyper androgenic symptoms and level of anxiety among young girls with Polycystic Ovarian Syndrome | Duplicate |  |
| 2015 | Lara, L. A.-//-Ramos, F. K.-//-Kogure, G. S.-//-Costa, R. S.-//-Silva de Sá, M. F.-//-Ferriani, R. A.-//-dos Reis, R. M. | Impact of Physical Resistance Training on the Sexual Function of Women with Polycystic Ovary Syndrome | Different study design | Case controlled |
| 2010 | Thomson, R. L.-//-Buckley, J. D.-//-Lim, S. S.-//-Noakes, M.-//-Clifton, P. M.-//-Norman, R. J.-//-Brinkworth, G. D. | Lifestyle management improves quality of life and depression in overweight and obese women with polycystic ovary syndrome | Different intervention |  |
| 2022 | Jiskoot, G.-//-Dietz de Loos, A.-//-Timman, R.-//-Beerthuizen, A.-//-Laven, J.-//-Busschbach, J. | Lifestyle treatment in women with polycystic ovary syndrome: predictors of weight loss and dropout | Different study design |  |
| 2020 | AlHussain, F.-//-AlRuthia, Y.-//-Al-Mandeel, H.-//-Bellahwal, A.-//-Alharbi, F.-//-Almogbel, Y.-//-Awwad, O.-//-Dala'een, R.-//-Alharbi, F. A. | Metformin Improves the Depression Symptoms of Women with Polycystic Ovary Syndrome in a Lifestyle Modification Program | Different intervention |  |
| 2016 | Thomson, R. L.-//-Buckley, J. D.-//-Brinkworth, G. D. | Perceived exercise barriers are reduced and benefits are improved with lifestyle modification in overweight and obese women with polycystic ovary syndrome: a randomised controlled trial | Different study design |  |
| 2017 | Arentz, S.-//-Smith, C.-//-Abbott, J.-//-Fahey, P.-//-Cheema, B.-//-Bensoussan, A. | Randomized controlled trial of combined lifestyle and herbal medicine in women with polycystic ovary syndrome | Abstract/Protocol/Opinion/Review |  |
| 2020 | Patel, V.-//-Menezes, H.-//-Menezes, C.-//-Bouwer, S.-//-Bostick-Smith, C. A.-//-Speelman, D. L. | Regular Mindful Yoga Practice as a Method to Improve Androgen Levels in Women With Polycystic Ovary Syndrome: A Randomized, Controlled Trial | Different study design |  |
| 2025 | Colonetti, L., Uggioni, M. L. R., Prestes, G. D. S., Stangherlin, L., Junior, J. C. D., Moura, R., Sipriano, E. D. S., Madeira, K., Cardoso, H. S., Ferraz, S. D., Baptista, M. M., Grande, A. J., Ceretta, L. B., da Rosa, M. I., & Colonetti, T. | Effects of carbohydrate reduced diet associated with strength training on clinical signs of women with polycystic ovary syndrome: Randomized clinical trial | Different study design | Different outcomes |
| 2024 | Dilimulati, D., Shao, X., Wang, L., Cai, M., Zhang, Y., Lu, J., Wang, Y., Liu, H., Kuang, M., Chen, H., Zhang, M., & Qu, S. | Efficacy of WeChat-Based Digital Intervention Versus Metformin in Women With Polycystic Ovary Syndrome: Randomized Controlled Trial | Different study design | No control group |
| 2025 | Jiang, L., Chen, Y., & Huang, M. | The impact of high-intensity interval training on insulin sensitivity and quality of life in women with overweight polycystic ovary syndrome | Different study design | Quality of life |
| 2024 | Hooshmandi, H., Ghadiri-Anari, A., Ranjbar, A. M., Fallahzadeh, H., Hosseinzadeh, M., & Nadjarzadeh, A. | Effects of licorice extract in combination with a low-calorie diet on obesity indices, glycemic indices, and lipid profiles in overweight/obese women with polycystic ovary syndrome (PCOS): a randomized, double-blind, placebo-controlled trial | Different intervention |  |
| 2025 | Nasiri, M., Monazzami, A., Alavimilani, S., & Asemi, Z. | Modulation of hormonal, metabolic, inflammatory and oxidative stress biomarkers in women with polycystic ovary syndrome following combined (resistant and endurance) training: a randomized controlled trail | Different study design | Different outcomes |
| 2025 | Chudzicka-Strugała, I., Kubiak, A., Banaszewska, B., Wysocka, E., Zwozdziak, B., Siakowska, M., Pawelczyk, L., & Duleba, A. J. | Six-month randomized, placebo controlled trial of synbiotic supplementation in women with polycystic ovary syndrome undergoing lifestyle modifications | Different study design | Different outcomes |
| 2025 | Panjrath, Y., Pathak, V. N., & Kumar, K. | Surya Namaskar as a Gender-sensitive Intervention: Addressing Social Physique Anxiety (SPA) Issues in Women with PCOS | Different intervention |  |
| 2024 | Kataoka, J., Stener-Victorin, E., Schmidt, J., & Larsson, I. | A prospective 12-month structured weight loss intervention in women with severe obesity and polycystic ovary syndrome: Impact of weight loss on eating behaviors | Different intervention | surveys |
| 2024 | Choudhry, D | TO find out the effect of aerobic exercise and yoga on musculoskeletal fitness, fatigue, quality of life and endocrine parameter in women with polycystic ovary syndrome | Abstract/Protocol/Opinion/Review | Different outcomes & protocol |
| 2025 | Chen, Y | Comparison of the Efficacy of Web-Based Supervised Progressive Resistance Exercise Versus Metformin on non-obese patients with polycystic ovary syndrome: an Open-Label, Randomized Controlled Trial | Abstract/Protocol/Opinion/Review | Different outcomes |
| 2024 | Pesonen, E., Farrahi, V., Brakenridge, C. J., Ollila, M. M., Morin-Papunen, L. C., Nurkkala, M., Jämsä, T., Korpelainen, R., Moran, L. J., Piltonen, T. T., & Niemelä, M. | 24-hour movement behaviours and cardiometabolic markers in women with polycystic ovary syndrome (PCOS): a compositional data analysis | Different study design | No RCT |
| 2025 | Aboelmagd, S., Saleh, M. S., Shehata, M. M. A., Dahi, A. A., Abo-zaid, N. A., & Hassan, E. S. | Response of menstrual irregularity and infertility-related stress to ultrasound cavitation combined with aerobic exercise in polycystic ovarian syndrome: double blinded randomized controlled trial. | Different study design | Different outcomes |
| 2024 | Nahidi F, Ramezani Tehrani F, Ghodsi D, Jafari M, Majd HA, Abdolahian S | The impact of a lifestyle promotion program on anthropometric and clinical manifestations in adolescents with polycystic ovarian syndrome: a randomized controlled trial | Different study design |  |
| 2025 | Partash, N., Ghamsari, S. R., Shirinbolagh, Z. E., & Ebrahimi, E. | The Relationship Between Nutritional Patterns and Physical Activity in Adolescents with Polycystic Ovary Syndrome. | Different study design |  |
| 2025 | Masters, M., & Grevstad, N. | Relationship between body mass index and quality of life, use of dietary and physical activity self-management strategies, and mental health in individuals with polycystic ovary syndrome. | Different intervention | Surveys |
