# Supplementary material for: Evaluating the efficacy of aerobic exercise as therapy for depression and anxiety in women with PCOS: a systematic review
Source: BMJ Open Sport Exerc Med. 2026 Jan 19;12(1):e002709. doi: 10.1136/bmjsem-2025-002709 (PMC12820835; doi:10.1136/bmjsem-2025-002709)
Supplement: online supplemental file 3 [file bmjsem-12-1-s003.pdf]

Table 2.

Summary of randomised controlled trials examining the effects of exercise programs on depression and anxiety in women with PCOS.

Study	Groups	Exercise program	Intensity	Duration (minutes)	Frequency x/week	Study duration (weeks)	Follow up	*Outcome changes* Anxiety %Δ	Depression %Δ	*Follow-up changes* Anxiety %Δ	Depression %Δ
Kogure et al., 2020	Continuous aerobic training	Aerobic training<br>Intensity Δ every week	65-80% HR_{max}	30 – 50	3	16	N/A	↓24.7	↓32.4	NA	NA
	Intermittent aerobic training	Aerobic training<br>Intensity Δ every 3 minutes						↓23.7	↓28.6		
	Control	No exercise training instructions	-	-				↓4.5	↓1.4		
Santos et al., 2022	HIIT training	HIIT training	95% HR_{max}	40 – 60	3	12	30 days	↓42.2	↓43.3	↓45.3	↔43.3
	Control	No exercise<br>Any preference	Moderate-intensity	150 min weekly				↓20	↑19.6	↑8	↑24.6
Stener-Victorin et al., 2013	Exercise training	Aerobic exercise<br>Any preference	70-80% HR_{max}	30	3	16	32 weeks	↑3.6	↑4.8	↑12.5	↑5.1
	Acupuncture	14 treatments over 16 weeks	Low frequency (2 Hz)		*varied			↓12.3	↓9	↓13.3	↓9.3
	Control	No exercise<br>Advised on the benefits of PA.	-	-				↓0.9	↑11.1	↓10.5	↑7.2

Abbreviations: Δ, change; *varied, 2x/week for 2 weeks then 1x/week for 6 weeks then 1x/every other week for 8 weeks; ↓, symptoms improved (reduced severity); ↑ symptoms worsened (increased severity); ↔, no change; PA, physical activity.
