# Supplementary material for: Evaluating the efficacy of aerobic exercise as therapy for depression and anxiety in women with PCOS: a systematic review
Source: BMJ Open Sport Exerc Med. 2026 Jan 19;12(1):e002709. doi: 10.1136/bmjsem-2025-002709 (PMC12820835; doi:10.1136/bmjsem-2025-002709)
Supplement: online supplemental material 1 [file bmjsem-12-1-s004.docx]

**Electronic Supplementary Material 3: Reporting Checklists.**

This file includes PRISMA compliance checklists for the abstract and the review.

Table of Contents

[Appendix 1. 2](#_Toc213407730)

[The PRISMA 2020 Abstract reporting checklist. 2](#_Toc213407731)

[Appendix 2. 3](#_Toc213407732)

[The PRISMA 2020 reporting checklist. 3](#_Toc213407733)

**Appendix 1.**

*The PRISMA 2020 Abstract reporting checklist.*

| **Section and Topic** | **Item #** | **Checklist item** | **Reported (Yes/No)** |
| --- | --- | --- | --- |
| **TITLE** | | |  |
| Title | 1 | Identify the report as a systematic review. | Yes |
| **BACKGROUND** | | |  |
| Objectives | 2 | Provide an explicit statement of the main objective(s) or question(s) the review addresses. | Yes |
| **METHODS** | | |  |
| Eligibility criteria | 3 | Specify the inclusion and exclusion criteria for the review. | Yes |
| Information sources | 4 | Specify the information sources (e.g. databases, registers) used to identify studies and the date when each was last searched. | Yes |
| Risk of bias | 5 | Specify the methods used to assess risk of bias in the included studies. | Yes |
| Synthesis of results | 6 | Specify the methods used to present and synthesise results. | Yes |
| **RESULTS** | | |  |
| Included studies | 7 | Give the total number of included studies and participants and summarise relevant characteristics of studies. | Yes |
| Synthesis of results | 8 | Present results for main outcomes, preferably indicating the number of included studies and participants for each. If meta-analysis was done, report the summary estimate and confidence/credible interval. If comparing groups, indicate the direction of the effect (i.e. which group is favoured). | Yes |
| **DISCUSSION** | | |  |
| Limitations of evidence | 9 | Provide a brief summary of the limitations of the evidence included in the review (e.g. study risk of bias, inconsistency and imprecision). | Yes |
| Interpretation | 10 | Provide a general interpretation of the results and important implications. | Yes |
| **OTHER** | | |  |
| Funding | 11 | Specify the primary source of funding for the review. | Yes |
| Registration | 12 | Provide the register name and registration number. | Yes |

**Appendix 2.**

*The PRISMA 2020 reporting checklist.*

The PRISMA 2020 reporting checklist

For checking that systematic review articles can be understood and used by everyone

| 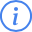 Note |
| --- |
| If you have not used a reporting guideline before, read about [how and why to use them](https:/resources.equator-network.org/about/reporting-guidelines.html) and check whether PRISMA 2020 is the [most applicable reporting guideline](https:/resources.equator-network.org/guidelines/prisma/index.html?#applicability) for your work.  Reporting guidelines are most useful when used early in research. When writing a manuscript or application, consider using the [Full Guidance](https:/resources.equator-network.org/guidelines/prisma/index.html) where you’ll see explanations and examples for each item.  After writing, demonstrate adherence by completing this checklist:   1. Specify where each item is described (see [Note 1](#sec-specify)). 2. Cite this checklist (See [Note 2](#sec-cite)). 3. Include your completed checklist as a supplement when submitting to a journal so that future readers can use it to find information. |

|  | Item Description | Location (or reason for not reporting) |
| --- | --- | --- |
| **Title and Abstract** |  |  |
| [1. Title](https:/resources.equator-network.org/guidelines/prisma/items/title.html) | Identify the report as a systematic review. | Page 1 |
| [2. Abstract](https:/resources.equator-network.org/guidelines/prisma/items/abstract.html) | Include all items from the *PRISMA 2020 for Abstracts* checklist. | Page 2 & 3 |
| **Introduction** |  |  |
| [3. Rationale](https:/resources.equator-network.org/guidelines/prisma/items/rationale.html) | Describe the rationale for the review in the context of existing knowledge. | Page 5 paragraph 4 |
| [4. Objectives](https:/resources.equator-network.org/guidelines/prisma/items/objectives.html) | Provide an explicit statement of the objective(s) or question(s) the review addresses. | Page 5 paragraph 4 |
| **Methods** |  |  |
| [5. Eligibility criteria](https:/resources.equator-network.org/guidelines/prisma/items/eligibility-criteria.html) | Specify the inclusion and exclusion criteria for the review and how studies were grouped for the syntheses. | Page 5 & 6 under **Eligibility criteria** |
| [6. Information sources](https:/resources.equator-network.org/guidelines/prisma/items/information-sources.html) | Specify all databases, registers, websites, organisations, reference lists, and other sources searched or consulted to identify studies. Specify the date when each source was last searched or consulted   - Specify the date when each source (such as database, register, website, organisation) wa… | Page 6 under **Information Sources** |
| [8. Search](https:/resources.equator-network.org/guidelines/prisma/items/search.html) | Present the full search strategies for all databases, registers, and websites, including any filters and limits used   - Provide the full line by line search strategy as run in each database with a sophisticated interface (such as Ovid), or the sequence of terms that were used to search si… | Page 4 – 6 in **electronic supplementary material (ESM) 1:** Appendix 1 - 3. |
| [8. Selection Process](https:/resources.equator-network.org/guidelines/prisma/items/selection-process.html) | Specify the methods used to decide whether a study met the inclusion criteria of the review, including how many reviewers screened each record and each report retrieved, whether they worked independently, and, if applicable, details of automation tools used in the process. | Page 6 under **Selection Process** |
| [9. Data collection process](https:/resources.equator-network.org/guidelines/prisma/items/data-collection-process.html) | Specify the methods used to collect data from reports, including how many reviewers collected data from each report, whether they worked independently, any processes for obtaining or confirming data from study investigators, and, if applicable, details of automation tools used in the process   - R… | Page 7 under **Data Collection process** |
| 10. Data Items |  |  |
| [10a. Outcomes](https:/resources.equator-network.org/guidelines/prisma/items/data-items-outcomes.html) | List and define all outcomes for which data were sought. Specify whether all results that were compatible with each outcome domain in each study were sought (for example, for all measures, time points, analyses), and, if not, the methods used to decide which results to collect.   - List and de… | Page 7 under **Data Items** |
| [10b. Other Variables](https:/resources.equator-network.org/guidelines/prisma/items/data-items-other-variables.html) | List and define all other variables for which data were sought (such as participant and intervention characteristics, funding sources). Describe any assumptions made about any missing or unclear information   - List and define all other variables for which data were sought. It may be sufficien… | Page 7 under **Data Items** |
| [11. Risk of bias in individual studies](https:/resources.equator-network.org/guidelines/prisma/items/risk-of-bias-in-individual-studies.html) | Specify the methods used to assess risk of bias in the included studies, including details of the tool(s) used, how many reviewers assessed each study and whether they worked independently, and, if applicable, details of automation tools used in the process.   - Specify the tool(s) (and version) u… | Page 7 under **Study risk of bias assessment** |
| [12. Effect measures](https:/resources.equator-network.org/guidelines/prisma/items/effect-measures.html) | Specify for each outcome the effect measure(s) (such as risk ratio, mean difference) used in the synthesis or presentation of results.   - Specify for each outcome or type of outcome (such as binary, continuous) the effect measure(s) (such as risk ratio, mean difference) used in the synthe… | Page 7 under **Effect Measures** |
| 13. Synthesis Methods |  |  |
| [13a. Deciding which studies were eligible for each synthesis](https:/resources.equator-network.org/guidelines/prisma/items/synthesis-methods-eligibility.html) | Describe the processes used to decide which studies were eligible for each synthesis (such as tabulating the study intervention characteristics and comparing against the planned groups for each synthesis described in item 5. | Page 6 under **Selection Process**  Page 7 under **Effect Measures** |
| [13b. Data preparation methods](https:/resources.equator-network.org/guidelines/prisma/items/synthesis-methods-data-preparation.html) | Describe any methods required to prepare the data for presentation or synthesis, such as handling of missing summary statistics or data conversions.   - Report any methods required to prepare the data collected from studies for presentation or synthesis, such as handling of missing summary… | Page 7 under **Data Collection Process**  Page 7 & 8 under **Synthesis Methods** |
| [13c. Methods for tabulating or displaying results](https:/resources.equator-network.org/guidelines/prisma/items/synthesis-methods-tabulating-or-displaying-results.html) | Describe any methods used to tabulate or visually display results of individual studies and syntheses   - Report chosen tabular structure(s) used to display results of individual studies and syntheses, along with details of the data presented. - Report chosen graphical methods used to v… | Page 7 & 8 under **Synthesis methods**  And  Page 9 **Table 1** **Overview of characteristics of randomised controlled trials in women with PCOS.**  and **Table 2** (submitted as a separate file, to be hyperlinked in the final publication). Title:  **Table 2** **Summary of randomised controlled trials examining the effects of exercise programs on depression and anxiety in women with PCOS.** |
| [13d. Synthesis methods](https:/resources.equator-network.org/guidelines/prisma/items/synthesis-methods-synthesis-methods.html) | Describe any methods used to synthesise results and provide a rationale for the choice(s). If meta-analysis was performed, describe the model(s), method(s) to identify the presence and extent of statistical heterogeneity, and software package(s) used.   - If statistical synthesis methods were used… | Page 7 & 8 under **Synthesis methods** |
| [13e. Methods for exploring heterogeneity](https:/resources.equator-network.org/guidelines/prisma/items/synthesis-methods-exploring-heterogeneity.html) | Describe any methods used to explore possible causes of heterogeneity among study results (such as subgroup analysis, meta-regression).   - If methods were used to explore possible causes of statistical heterogeneity, specify the method used (such as subgroup analysis, meta-regression).   -… | N/A no heterogeneity analysis due to the use of varied mental health questionnaires reduced comparability across studies, and heterogeneity in interventions. |
| [13f. Sensitivity analyses](https:/resources.equator-network.org/guidelines/prisma/items/synthesis-methods-sensitivity-analyses.html) | Describe any sensitivity analyses conducted to assess robustness of the synthesised results.   - If sensitivity analyses were performed, provide details of each analysis (such as removal of studies at high risk of bias, use of an alternative meta-analysis model). - If any sensitivity an… | N/A no sensitivity analysis. |
| [14. Reporting bias assessment](https:/resources.equator-network.org/guidelines/prisma/items/reporting-bias-assessment.html) | Describe any methods used to assess risk of bias due to missing results in a synthesis (arising from reporting biases)   - Specify the methods (tool, graphical, statistical, or other) used to assess the risk of bias due to missing results in a synthesis (arising from reporting biases). - … | Page 7 under **Study risk of bias assessment**  And Page 2 and 3 in **ESM 1:** Table 1 and 2 for Risk of bias tables for anxiety and depression**.** |
| [15. Certainty assessment](https:/resources.equator-network.org/guidelines/prisma/items/certainty-assessment.html) | Describe any methods used to assess certainty (or confidence) in the body of evidence for an outcome   - Specify the tool or system (and version) used to assess certainty in the body of evidence. - Report the factors considered (such as precision of the effect estimate, consistency of f… | Page 17 under **Strengths & limitations** |
| **Results** |  |  |
| 16. Study Selection |  |  |
| [16a. Results of the search and selection process](https:/resources.equator-network.org/guidelines/prisma/items/study-selection-search-results.html) | Describe the results of the search and selection process, from the number of records identified in the search to the number of studies included in the review, ideally using a flow diagram | Page 10 under **Study selection**  and  **Figure 1 Flow chart of the study selection process** (submitted as a separate file, to be hyperlinked in the final publication). |
| [16b. Excluded studies](https:/resources.equator-network.org/guidelines/prisma/items/study-selection-excluded-studies.html) | Cite studies that might appear to meet the inclusion criteria, but which were excluded, and explain why they were excluded.   - Cite studies that might appear to meet the inclusion criteria, but which were excluded, and explain why they were excluded. | Page 10 under **Study selection**  and  **Figure 1 Flow chart of the study selection process** (submitted as a separate file, to be hyperlinked in the final publication)  and  Page 4 in **ESM 2: Supplementary Table 3.** |
| [17. Study characteristics](https:/resources.equator-network.org/guidelines/prisma/items/study-characteristics.html) | Cite each included study and present its characteristics.   - Cite each included study. - Present the key characteristics of each study in a table or figure (considering a format that will facilitate comparison of characteristics across the studies). | Page 10 under **Study characteristics**  Page 9 **Table 1 Overview of characteristics of randomised controlled trials in women with PCOS.** |
| [18. Risk of bias in studies](https:/resources.equator-network.org/guidelines/prisma/items/risk-of-bias-in-studies.html) | Present assessments of risk of bias for each included study   - Present tables or figures indicating for each study the risk of bias in each domain/component/item assessed and overall study-level risk of bias. - Present justification for each risk of bias judgment—for example, in t… | Page 11 under **Risk of bias in studies**  And page 2 and 3 in **ESM 1: Table 1 and 2** for Risk of bias tables for anxiety and depression**.** |
| [19. Results of individual studies](https:/resources.equator-network.org/guidelines/prisma/items/results-of-individual-studies.html) | For all outcomes, present for each study (*a*) summary statistics for each group (where appropriate) and (*b*) an effect estimate and its precision (such as confidence/credible interval), ideally using structured tables or plots   - For all outcomes, irrespective of whether statistical synthesis w… | Page 11, 13 under **Outcomes** with subheadings: **Depression** and **Anxiety**  **Table 2 Summary of randomised controlled trials examining the effects of exercise programs on depression and anxiety in women with PCOS.** (submitted as a separate file, to be hyperlinked in the final publication). |
| 20. Results of Synthesis |  |  |
| [20a. Summary of studies](https:/resources.equator-network.org/guidelines/prisma/items/results-of-syntheses-summary-of-studies.html) | For each synthesis, briefly summarise the characteristics and risk of bias among contributing studies.   - Provide a brief summary of the characteristics and risk of bias among studies contributing to each synthesis (meta-analysis or other). The summary should focus only on study character… | Page 10, 11 under **Intervention characteristics**  Page 11 under **Risk of bias in studies**  And page 2 and 3 in **ESM 1: Table 1 and 2** for Risk of bias tables for anxiety and depression**.** |
| [20b. Statistical results](https:/resources.equator-network.org/guidelines/prisma/items/results-of-syntheses-statistical-results.html) | Present results of all statistical syntheses conducted. If meta-analysis was done, present for each the summary estimate and its precision (such as confidence/credible interval) and measures of statistical heterogeneity. If comparing groups, describe the direction of the effect.   - Report results… | Page 11, 13 under **Outcomes** with subheadings: **Depression** and **Anxiety**  And  **Table 2 Summary of randomised controlled trials examining the effects of exercise programs on depression and anxiety in women with PCOS.** (submitted as a separate file, to be hyperlinked in the final publication).  And  Page 2 and 3 in **ESM 1: Table 1 and 2** for Risk of bias tables for anxiety and depression  And  Page 2 and 3 in **ESM 2:** data extraction sheets for anxiety and depression. |
| [20c. Heterogeneity](https:/resources.equator-network.org/guidelines/prisma/items/results-of-syntheses-heterogeneity.html) | Present results of all investigations of possible causes of heterogeneity among study results.   - If investigations of possible causes of heterogeneity were conducted:   - present results regardless of the statistical significance, magnitude, or direction of effect modification. … | N/A no heterogeneity analysis due to the use of varied mental health questionnaires reduced comparability across studies, and heterogeneity in interventions. |
| [20d. Sensitivity analyses](https:/resources.equator-network.org/guidelines/prisma/items/results-of-syntheses-sensitivity-analyses.html) | Present results of all sensitivity analyses conducted to assess the robustness of the synthesised results   - If any sensitivity analyses were conducted:   - report the results for each sensitivity analysis.   - comment on how robust the main analysis was given the results of all… | N/A no sensitivity analysis. |
| [21. Risk of reporting biases in syntheses](https:/resources.equator-network.org/guidelines/prisma/items/risk-of-reporting-biases-in-syntheses.html) | Present assessments of risk of bias due to missing results (arising from reporting biases) for each synthesis assessed   - Present assessments of risk of bias due to missing results (arising from reporting biases) for each synthesis assessed. - If a tool was used to assess risk of bias due … | Page 11 under **Risk of bias in Studies**  Page 2 and 3 in **ESM 1: Table 1 and 2** for Risk of bias tables for anxiety and depression |
| [22. Certainty of evidence](https:/resources.equator-network.org/guidelines/prisma/items/certainty-of-evidence.html) | Present assessments of certainty (or confidence) in the body of evidence for each outcome assessed   - Report the overall level of certainty in the body of evidence (such as high, moderate, low, or very low) for each important outcome. - Provide an explanation of reasons for rating down (or… | Page 11 under **Risk of bias in Studies**  Page 17 under **Strengths & limitations** |
| **Discussion** |  |  |
| 23. Discussion |  |  |
| [23a. General interpretation of the results](https:/resources.equator-network.org/guidelines/prisma/items/discussion-general-interpretation.html) | Provide a general interpretation of the results in the context of other evidence   - Provide a general interpretation of the results in the context of other evidence. | Page 13 under **Discussion** and under subheadings **Exercise benefits for depression and anxiety** page 14-15**;**  **Exercise benefits of supervised aerobic exercise** page 16-17**.** |
| [23b. Limitations of included evidence](https:/resources.equator-network.org/guidelines/prisma/items/discussion-limitations-of-included-evidence.html) | Discuss any limitations of the evidence included in the review   - Discuss any limitations of the evidence included in the review. | Page 17 under **Strengths & limitations** |
| [23c. Limitations of the review processes](https:/resources.equator-network.org/guidelines/prisma/items/discussion-limitations-of-review-process.html) | Discuss any limitations of the review processes used   - Discuss any limitations of the review processes used and comment on the potential impact of each limitation. | Page 17 under **Strengths & limitations** |
| [23d. Implications](https:/resources.equator-network.org/guidelines/prisma/items/discussion-implications.html) | Discuss implications of the results for practice, policy, and future research   - Discuss implications of the results for practice and policy. - Make explicit recommendations for future research. | Page 17 under **future directions** |
| **Other Information** |  |  |
| 24. Registration and Protocol |  |  |
| [24a. Registration](https:/resources.equator-network.org/guidelines/prisma/items/registration-and-protocol-registration.html) | Provide registration information for the review, including register name and registration number, or state that the review was not registered   - Provide registration information for the review, including register name and registration number, or state that the review was not registered. | Page 2-3 under PROSPERO identifier  and  Page 5 first paragraph under **Methods**.  PROSPERO identifier: CRD42023408190  https://www.crd.york.ac.uk/PROSPERO/view/CRD42023408190 |
| [24b. Protocol](https:/resources.equator-network.org/guidelines/prisma/items/registration-and-protocol-protocol.html) | Indicate where the review protocol can be accessed, or state that a protocol was not prepared   - Indicate where the review protocol can be accessed (such as by providing a citation, DOI, or link) or state that a protocol was not prepared. | Page 2-3 under PROSPERO identifier  and  page 5 first paragraph under **methods**.  PROSPERO identifier: CRD42023408190  https://www.crd.york.ac.uk/PROSPERO/view/CRD42023408190 |
| [24c. Amendments](https:/resources.equator-network.org/guidelines/prisma/items/registration-and-protocol-amendments.html) | Describe and explain any amendments to information provided at registration or in the protocol   - Report details of any amendments to information provided at registration or in the protocol, noting: (*a*) the amendment itself, (*b*) the reason for the amendment, and (*c*) the stage of the… | N/A no amendments and stated on page 5 under **methods 1^st^ paragraph** |
| [25. Support](https:/resources.equator-network.org/guidelines/prisma/items/support.html) | Describe sources of financial or non-financial support for the review, and the role of the funders or sponsors in the review   - Describe sources of financial or non-financial support for the review, specifying relevant grant ID numbers for each funder. If no specific financial or non-fina… | Page 3 under **Funding** |
| [26. Competing Interests](https:/resources.equator-network.org/guidelines/prisma/items/competing-interests.html) | Declare any competing interests of review authors   - Disclose any of the authors’ relationships or activities that readers could consider pertinent or to have influenced the review. - If any authors had competing interests, report how they were managed for particular review processes. | Page 18 under **Competing interests** |
| [27. Availability of data, code, and other materials](https:/resources.equator-network.org/guidelines/prisma/items/availability-of-materials.html) | Report which of the following are publicly available and where they can be found: template data collection forms; data extracted from included studies; data used for all analyses; analytic code; any other materials used in the review   - Report which of the following are publicly available: templa… | Page 3 under **Data availability statement**  And available at OSF: <https://osf.io/3ctdf/>. |
